# Evaporation-controlled dripping-onto-substrate (DoS) extensional rheology of viscoelastic polymer solutions

**DOI:** 10.1038/s41598-022-08448-x

**Published:** 2022-03-18

**Authors:** Benjamin P. Robertson, Michelle A. Calabrese

**Affiliations:** Department of Chemical Engineering and Materials Science, University of Minnesota, Minneapolis, 55455 USA

**Keywords:** Fluid dynamics, Techniques and instrumentation, Soft materials, Techniques and instrumentation, Chemical engineering

## Abstract

Extensional flow properties of polymer solutions in volatile solvents govern many industrially-relevant coating processes, but existing instrumentation lacks the environment necessary to control evaporation. To mitigate evaporation during dripping-onto-substrate (DoS) extensional rheology measurements, we developed a chamber to enclose the sample in an environment saturated with solvent vapor. We validated the evaporation-controlled DoS device by measuring a model high molecular weight polyethylene oxide (PEO) in various organic solvents both inside and outside of the chamber. Evaporation substantially increased the extensional relaxation time $\lambda _{E}$ for PEO in volatile solvents like dichloromethane and chloroform. PEO/chloroform solutions displayed an over 20-fold increase in $\lambda _{E}$ due to the formation of an evaporation-induced surface film; evaporation studies confirmed surface features and skin formation reminiscent of buckling instabilities commonly observed in drying polymer solutions. Finally, the relaxation times of semi-dilute PEO/chloroform solutions were measured with environmental control, where $\lambda _{E}$ scaled with concentration by the exponent $m=0.62$. These measurements validate the evaporation-controlled DoS environment, and confirm that chloroform is a good solvent for PEO, with a Flory exponent of $\nu =0.54$. Our results are the first to control evaporation during DoS extensional rheology, and provide guidelines establishing when environmental control is necessary to obtain accurate rheological parameters.

## Introduction

Extensional flows play an important role in the transfer^1^, deposition, and breakup of low-viscosity volatile fluids in industrial processes from coating^2,3^ and inkjet printing^4,5^ to fuel injection^6^. Complex flows like forward roll coating^3^ and spraying^7,8^ generate droplets via the extension and breakup of filaments. Volatile organic solvents are often used in these industrial processes to dissolve formulation components and enable facile drying of coatings once deposited^9–11^. In addition to the applied processing parameters, the fluid viscoelasticity and corresponding extensional rheological properties dictate the fraction of fluid that breaks up into droplets, the timescales for breakup, and the droplet size distribution^7^. These rheological parameters can thus be used to quantify the coatability and sprayability of macromolecular solutions^12–14^, which when paired with the rate of solvent evaporation, largely dictate the coating quality^15^. Although properties like zero-shear viscosity play a role in governing these flows, counterparts like extensional viscosity determine the final breakup of fluid droplets. Additionally, the extensional viscosity in dilute polymer solutions is often orders of magnitude larger than the shear viscosity^2,16^, a property which can be detrimental in coating and printing applications. As extensional flows impart deformations that disrupt the structure of complex fluid elements like polymer coils more substantially than in the analogous shear flows^17,18^, extensional rheology may also be more indicative of performance in coating processes for low-viscosity fluids. The prevalence of extensional flow in industrial processes and its strong impact on polymer conformation thus makes extensional rheology particularly useful in measuring fundamental material properties of complex fluids and in guiding sample formulation prior to scale-up.

Rheological parameters like extensional viscosity and relaxation time ($\lambda _{E}$) that are useful in characterizing these flows cannot be predicted by shear behavior alone^2,19^. While uniaxial extensional flow can be generated by a microfluidic device^20^ or a jet^16,21^, both of these techniques use custom-fabricated devices, requiring a priori knowledge of fluid properties like extensional viscosity to determine the extension rates that occur. The extension rate of a fluid in a microfluidic device or a jet is dependent on the size of the custom-built channel or nozzle^21,22^, so extraction of material properties can be a complicated, iterative process. Furthermore, these techniques often produce mixed shear and extensional flows, which makes distinguishing the specific impact of extensional flow challenging.

A technique to generate a more well-defined extensional flow in low-viscosity fluids is capillary-driven thinning and breakup of a stretched liquid bridge. Here, a liquid bridge self-thins and breaks up in the absence of active external forces^2,23^. Depending on the balance of inertial, viscous, elastic, and capillary forces on the thinning liquid bridge, the minimum radius of the liquid bridge, *R*, evolves in time following scaling laws corresponding to a range of flow regimes. The dimensionless Ohnesorge number, *Oh*, describes the balance of viscous to inertial and surface tension forces, given by:

1 $$\begin{aligned} Oh=\frac{\eta _{0}}{\sqrt{\rho \sigma R_{0}}} \end{aligned}$$

where $\eta _{0}$, $\rho$, and $\sigma$ are the solution zero-shear viscosity, density and surface tension respectively, and $R_{0}$ is the initial radius of the liquid bridge, often approximated by the radius of the nozzle or plates used to generate the bridge. For low-viscosity fluids ($Oh<1$), thinning is expressed as a balance between inertial and capillary forces, known as inertio-capillary (IC) thinning. When $Oh>1$, viscous forces become important and visco-capillary (VC) thinning occurs. For fluids with significant elasticity such as polymer solutions, elastic forces can begin to dominate over inertial and viscous forces during thinning, causing a transition into the elasto-capillary (EC) regime. Expressions that mathematically describe the thinning phenomena in each regime can subsequently be used to extract a range of rheological and processing parameters, like the extensional relaxation time $\lambda _{E}$ or the “breakup” or pinch-off time at which the filament ruptures, $t_{b}$^2,24–26^.

Mathematical descriptions of capillary self-thinning phenomena depend on assumptions of fixed endpoints, homogeneous composition of the liquid bridge, and steady thinning unperturbed by oscillations of the free surface. The unstable liquid bridge is typically formed in an instrument like the Capillary Breakup Extensional Rheometer (CaBER)^27–29^ via the rapid separation of two plates. However, low-viscosity samples often thin on timescales similar to the time required for plate separation, preventing formation of a self-thinning liquid bridge^30^. Furthermore, rapid plate separation can cause severe inertial effects^2,16,31,32^, which are exacerbated by the low surface tensions of solutions formed in organic solvents. Evaporation is a further concern in measuring volatile systems^5,33^, given that the CaBER plates are not well-sealed to the ambient environment. While custom-built CaBER instruments have introduced chambers with better sealing for temperature control^34,35^, these studies did not utilize the chambers for controlling evaporation. Previous work by Sousa and coworkers^33,36^ incorporates true environmental control by suspending the aqueous samples in an oil bath, but this method introduces an oil–water interface which further affects liquid bridge thinning^37^ and is unsuitable for samples miscible in oil. Similar to CaBER, a sealed micro-filament rheometer has been used to measure volatile pressure-sensitive adhesive fluids and a 1D Newtonian model was developed to predict filament thinning in the presence of volatile solvent^14^. Unfortunately, the model and experiments did not agree well, especially for more volatile samples.

An alternative to CaBER is the recently-developed dripping-onto-substrate (DoS) rheometry technique^23,32^. In the DoS method, the unstable liquid bridge is generated via the slow dripping of a single drop onto a substrate. This technique enables measurement of complex fluids in small volumes ($\sim$ 10 $\upmu$L), reducing use of expensive sample components, mitigating pre-deformation, and minimizing inertial effects, which allows for the measurement of low surface-tension, low-viscosity systems. By directly mounting the syringe above the substrate^38^, contact between the sample and excess tubing used in the standard DoS instrumentation^23,32^ can be eliminated, preventing leaching issues from the use of organic solvents and further reducing sample volume. However, the small volume and high surface area-to-volume ratio of the drop exacerbates evaporation effects in volatile systems, and DoS instrumentation to-date has not employed true environmental control^5,23,38,39^.

While most DoS experiments to-date focus on aqueous systems, recent work demonstrates use of the DoS technique to measure complex fluids in solvents with moderate boiling points ($T_{BP}\ge 65\,^{\circ }$C) like methyl ethyl ketone^5^, methanol^40^, and ethyl acetate^41^. These studies attempted to limit evaporation by performing trials quickly to minimize the time that the sample contacts the air. Recent work by Merchiers and coworkers used a sealed environment to slow evaporation in acetonitrile/water solutions, albeit without a solvent reservoir to saturate the environment^42^. However, none of these works quantified or estimated evaporation effects on the pendant drop. As the evaporation rate is a central consideration in selecting processing parameters for coating processes incorporating volatile solvents^43,44^, these volatile coatings can only be studied by controlling evaporation to the environment. Furthermore, without a method of true environmental control, the effect of evaporation on extension cannot be easily separated from the effect of flow rate^5,41^ or of solvent quality^42^. Currently, evaporation effects are best studied by repeating trials at varying exposure times, similar to work by Tripathi and McKinley on hygroscopic glycerol solutions^45^.

As such, capillary thinning of dilute polymer solutions has yet to be characterized in a geometry with a free surface at which the rate of evaporation can be controlled, so capillary thinning extensional rheometry has largely been limited to studying solvents with relatively high boiling points. In addition, there has been no study of the coupling effects of evaporation and extension during DoS measurements. Evaporation can concentrate the sample or cause surface inhomogeneities like films, similar to those observed in fiber spinning^46–48^ and in sessile drops^49,50^, which would affect the thinning behavior of the liquid bridge even in the dilute regime.

To accurately measure dripping-onto-substrate (DoS) extensional rheology in complex fluids dissolved in volatile organic solvents free of evaporation effects, we developed a direct-mount DoS device^38^, which was further adapted to enclose the liquid bridge in an environmental control chamber to control evaporation. To validate the instrument and examine evaporative effects, a high-molecular weight polyethylene oxide (PEO, $M_{W}= 10^{6}$ g/mol) sample was examined in solvents of varying quality and volatility. PEO was selected as a model system due to its extensive characterization in dilute and semi-dilute aqueous solutions via capillary thinning methods (CaBER and DoS)^2,23,51–54^. Here, the elasto-capillary (EC) thinning behavior^2,23,52–54^ of PEO was compared in the freely-evaporating versus environmentally-controlled configurations of the instrument. Using a combination of DoS, surface tension measurements, shear rheology, and evaporation studies, we demonstrate the utility of evaporation-controlled DoS for measuring accurate extensional rheology of polymers dissolved in highly volatile solvents. In addition to identifying evaporation-induced concentration changes, these studies also reveal that surface films can form during DoS at ambient conditions. By assessing the impact of solvent evaporation on extension, we validate our technique for evaporation-controlled DoS and establish guidelines for when environmental control during DoS extensional rheology is required for accurate measurements.

## Results

### DoS environmental chamber development

**Figure 1:**
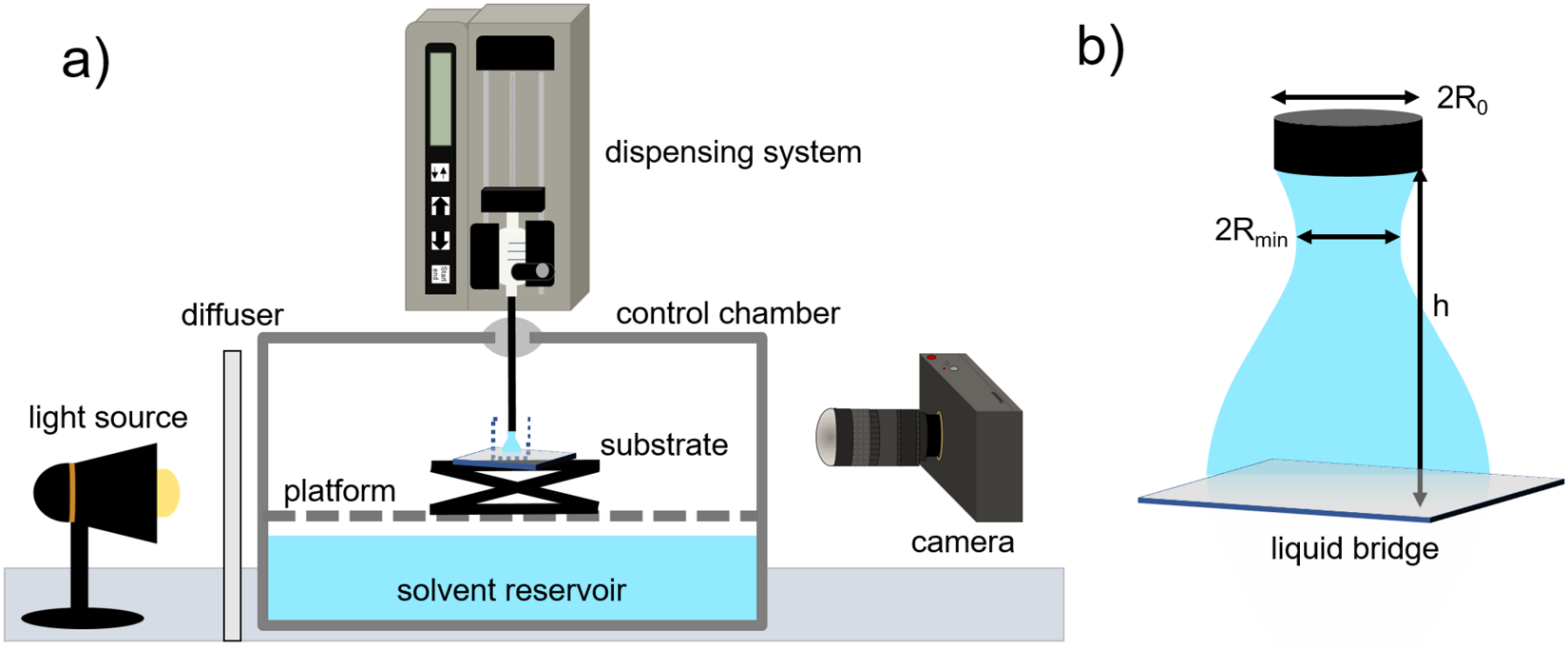
Schematic of the evaporation-controlled DoS instrument with sacrificial solvent reservoir and environmental control chamber, adapted from Lauser et al.^38^ (see SI.2); set-up adapted from Dinic and Sharma^23^. To perform DoS measurements, the syringe pump is used to extrude a droplet from a needle with radius $R_{0}$, which then forms a liquid bridge with height *h* upon contact with the substrate. A high-speed camera captures the evolution of the liquid bridge minimum radius *R* as the bridge thins in time. The environmental control chamber provides an atmosphere enriched in solvent vapor around the drop to prevent evaporation during this thinning process.

The dripping-onto-substrate device consists of a dispensing system to extrude the drop which forms the liquid bridge and an imaging system to precisely measure the thinning profile of the bridge at high speed (Fig. 1; see “Methods” section).

To modify the DoS instrument to provide an atmosphere enriched in solvent vapor, a glass chamber was altered by adding a self-healing septum, silicone sealant, and screws to attach the lid clamps and serve as internal fasteners. A custom platform was designed to fit in the chamber, 3D printed in nylon, and attached to screws on the inside to support a miniature lab jack above the solvent reservoir. To make evaporation-controlled DoS measurements (Fig. 1), the chamber is placed beneath the syringe pump on anti-vibration slats to minimize mechanical interference, with the needle aligned with the septum. After filling the reservoir with solvent, the chamber atmosphere is allowed to saturate with solvent vapor for $\ge$ 45 min prior to measurement. Evaporation studies on pendant drops of PEO in four solvents confirm that 45 min is sufficient to mitigate evaporation for timescales longer than required to perform the DoS measurement (SI.10); additional DoS trials after 2h equilibration confirm that $\lambda _{E}$ is not impacted by equilibration time after 45 min (SI.11).

### Validation of evaporation-controlled DoS device with PEO in various solvents

**Figure 2:**
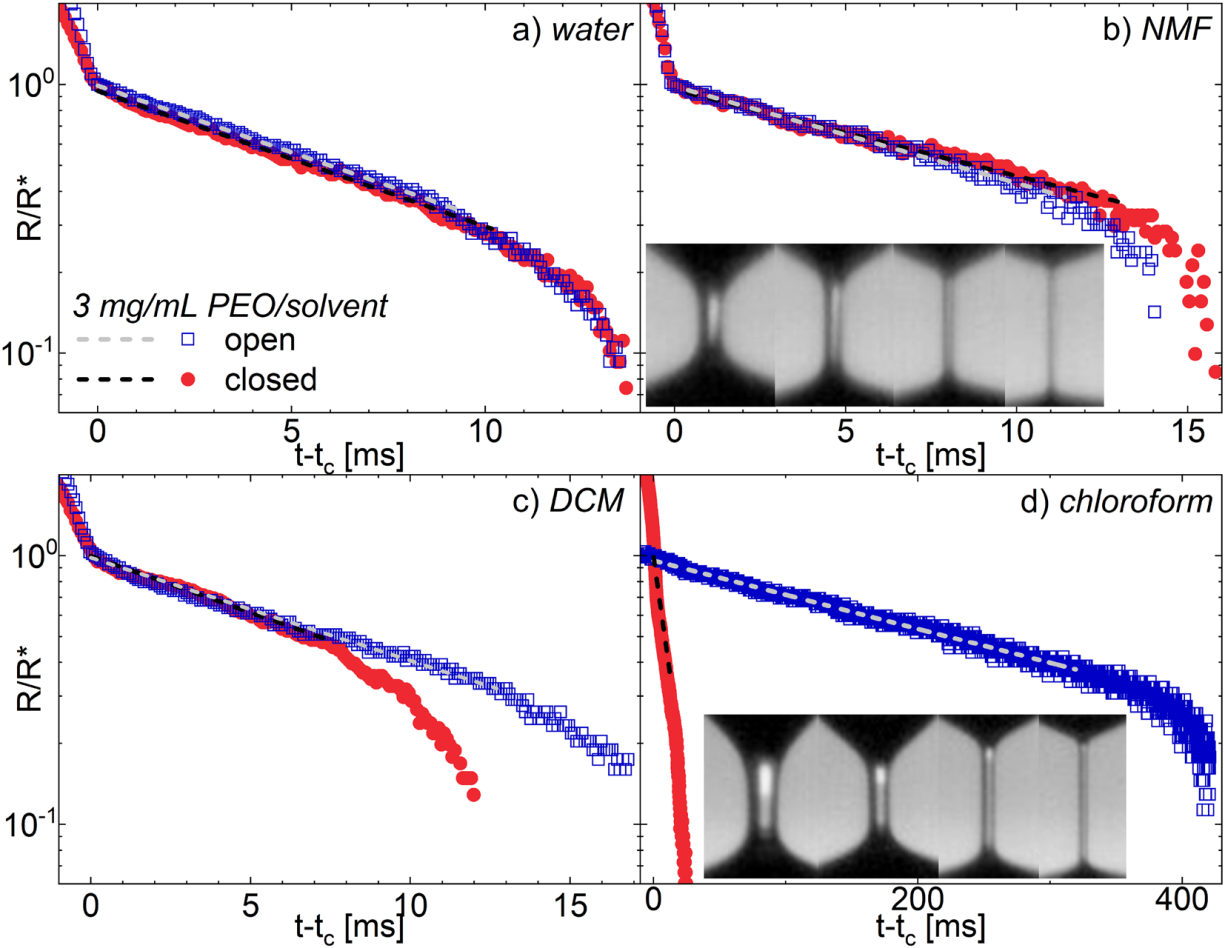
Representative evaporation-controlled DoS capillary thinning measurements of 3 mg/mL PEO in: (**a**) water, (**b**) NMF, (**c**) DCM, and (**d**) chloroform. Insets in (**b**) and (**d**) show 2D images of the thinning inside of the chamber at (from L to R) $R/R^{*}= 1$ (onset of EC regime), 0.7, 0.4 and 0.1. (**a**,**b**) As expected, no statistically significant difference in $\lambda _{E}$ is observed for low volatility solvents (water, NMF); see Table 1. (**c**,**d**) In more volatile solvents (chloroform, DCM), pronounced differences in the thinning profiles and $\lambda _{E}$ result between trials taken inside versus outside the environmental control chamber

Capillary thinning and evaporation measurements of PEO in solvents of different quality and volatility demonstrate that the environmental control chamber is highly effective in limiting evaporation (Fig. 2). Solvent quality was estimated via shear rheology (Table 1, SI.8) and relative energy density (RED) values calculated from Hansen solubility parameters^55^; smaller values indicate better solvent quality. Here, RED $\le$ 1 for each solvent with PEO, indicating fairly good solvent quality. Solvents include chloroform, dichloromethane (DCM), water, and N-methylformamide (NMF), with boiling points ranging from 40 to 183 $^{\circ }$C (Table 1). Figure 2 shows representative DoS trials and fits when the drop is enclosed inside of the chamber ($\bullet$ symbol) and when the drop is exposed to ambient conditions ($\square$ symbol, $\sim$ 23 $^{\circ }$C, 40% relative humidity); 2D images during thinning in the closed configuration are shown inset for solutions experiencing the least (PEO/NMF) and most significant (PEO/chloroform) evaporation effects. For raw data from all trials and reproducibility, see SI.13. Representative radial evolution profiles are nearly identical between the open and closed configurations for water and NMF, the two least volatile solvents (Fig. 2a,b); however, significant deviations in the thinning profiles are observed for PEO in DCM and chloroform (Fig. 2c,d), where trials performed without environmental control show substantially longer extensional relaxation times ($\lambda _{E}$) and filament lifetimes ($t_{b}$).

**Table 1:** Average $\lambda _{E}$ and *n* values for 3 mg/mL PEO solutions; ‘open’ indicates no environmental control whereas ‘closed’ occurs with the chamber sealed. Open and closed $\lambda _{E}$ values are statistically equal for water and NMF, whereas chloroform and DCM solutions exhibit longer $\lambda _{E}$ with the chamber open. Chloroform and DCM are more volatile (higher vapor pressures $P_{vap}$ and lower boiling points $T_{BP}$), less viscous ($\eta _{s}$), and form lower surface tension ($\sigma _{solution}$) solutions than water and NMF; specific viscosities, $\eta _{sp}$, are also reported. All three organic solvents are predicted to be better solvents than water for PEO, given relative energy density (RED) values calculated from Hansen solubility parameters (SI.6) and shear rheology (SI.8).

Solvent	$\lambda _{closed}$ (ms)	$\lambda _{open}$ (ms)	$n_{closed}$	$P_{vap}$ (bar)	$T_{BP}$ ($^{\circ }$C)	$\eta _{s}$ (mPa s)	$\eta _{sp}$	$\sigma _{solution}$ (mN/m)	RED
Water	2.87 ± 0.29	3.04 ± 0.24	0.67 ± 0.03	3.2 × 10^−2^	100	0.89	3.7	62 ± 1	1.03
NMF	3.94 ± 0.37	3.96 ± 0.43	0.66 ± 0.03	3.4 × 10^−4^	183	1.68	4.2	37 ± 1	0.74
DCM	3.35 ± 0.17	4.65 ± 1.25	0.67 ± 0.03	5.7 × 10^−1^	40	0.41	9.1	25 ± 2	0.67
chloroform	4.41 ± 0.27	93.2 ± 79.2	0.66 ± 0.02	2.6 × 10^−1^	61	0.54	9.9	29 ± 1	0.82

All PEO solutions measured with environmental control display two distinct flow regimes; conversely, freely evaporating PEO/chloroform shows a broad transition between the flow behavior at early times and at long times. Given the low solution viscosities and PEO content (3 mg/mL), PEO solutions are expected to initially display inertiocapillary (IC) thinning ($Oh\ll 1$, Eq. (1)) prior to transitioning to an elastocapillary (EC) regime (SI.8). The IC regime is governed by the Rayleigh time^2,56^ ($t_{R}=\sqrt{\rho R_{0}^{3}/\sigma }$) for a fluid with density $\rho$ and surface tension $\sigma$. The evolution of the minimum radius *R* from its initial value $R_{0}$ during IC thinning is described by^2,24,25,57^:

2 $$\begin{aligned} \frac{R}{R_{0}}=\alpha \left( \frac{t_{f}-t}{t_{{R}}}\right) ^{\frac{2}{3}} \end{aligned}$$

where $\alpha$ is a constant and $t_{f}$ is the pinch-off time for an inviscid fluid. In the initial flow regime ($t-t_{c}<0$ in Fig. 2), PEO self-thins in all solvents via a power law scaling following the characteristic value of $n=2/3$ for inertiocapillary thinning (Table 1). See SI.15 for data plotted on a $t_{f}-t$ scale, individual trial data, and IC fits.

Following the initial IC regime, pronounced EC thinning occurs in all PEO solutions measured both with and without environmental control. The EC regime is defined by the extensional relaxation time of the polymer chains in solution, $\lambda _{E}$. When this relaxation can be described reasonably well by a single exponential decay function^2,26,58^, the liquid bridge thins as:

3 $$\begin{aligned} \frac{R}{R_{0}}\approx \left( \frac{GR_{0}}{2\sigma }\right) ^{\frac{1}{3}}\exp \left( \frac{-(t-t_{c})}{3\lambda _{E}}\right) \end{aligned}$$

where *G* is the elastic modulus and $t_{c}$ corresponds to the onset of the EC regime. Here, $(GR_{0}/2\sigma )^{1/3}$ is treated as a fitting constant, as is commonly done^23,38^. Note that in Fig. 2, *R* is instead normalized to $R^{*}$, the radius at $t_{c}$, to enable straightforward comparison between solutions.

The extracted extensional relaxation times for PEO solutions in water are statistically equal for measurements taken inside and outside of the chamber (Fig. 2a, Tables 1 and S1), and are in quantitative agreement with prior DoS results^23,38^. These prior studies did not incorporate environmental control, suggesting that ambient conditions are sufficient for measuring dilute aqueous polymers via DoS. Likewise, no statistically significant differences are observed between open and closed trials for solutions in NMF (Fig. 2b, SI.3), a solvent with lower volatility than water (Table 1). Following Eq. (3), the shallower slope in the fit region for PEO/NMF versus PEO/water indicates that PEO in NMF exhibits longer relaxation times ($\lambda _{E}=$ $3.9 \pm 0.4$ ms vs. $2.9 \pm 0.3$ ms, respectively). While these two solutions have similar specific viscosities, $\eta _{sp}=\frac{\eta _{0}-\eta _{s}}{\eta _{s}}$, the zero-shear viscosity for PEO/NMF is over twice that of PEO/water. The longer $\lambda _{E}$ in NMF is thus impacted by both its higher viscosity and better solvent quality for PEO, based on rheology (SI.8.5) and RED values (Table 1). For the semi-dilute, unentangled polymer solutions here (see SI.7–SI.8)^59^:

4 $$\begin{aligned} \eta _{sp} \approx \left( \frac{\phi }{\phi ^{*}}\right) ^{x} \approx N\phi ^{x} \end{aligned}$$

where *N* is the number of Kuhn segments, $\phi$ is the polymer volume fraction, and $\phi ^{*}$ is the volume fraction at the overlap concentration. The exponent $x =\frac{1}{3\nu -1}$ where $\nu$ is the Flory exponent, ranging from 0.5 (theta solvent, $x=$ 2) to 0.588 (good solvent, $x=$ 1.3)^59–61^. The marginally higher $\eta _{sp}$ in PEO/NMF versus PEO/water is thus likely a tradeoff between a lower *x*-value and a higher $\phi$ relative to $\phi ^{*}$ in PEO/NMF.

However, when PEO is dissolved in more volatile solvents like chloroform and DCM, strong differences in extensional flow parameters are observed due to evaporation at ambient conditions. For PEO in DCM, the filament lifetime $t_{b}$, or the time at which the filament breaks up, increases by roughly 40% when capillary thinning is performed outside of the chamber (Fig. 2c). The resulting relaxation time is also 40% longer for trials measured in ambient conditions than for those extracted from analogous measurements incorporating environmental control ($\lambda _{E}=$ $4.7 \pm 1.2$ ms vs. $3.4 \pm 0.2$ ms), likely due to higher solution concentration resulting from solvent evaporation (SI.12). The wider uncertainty associated with $\lambda _{E}$ for trials outside of the chamber stems from large variation in the radial decay profiles between repeated trials (see SI.13). This variation is likely driven by non-uniform evaporative effects during thinning due to the lack of controlled environment.

Evaporative effects during thinning are most significant for PEO in chloroform (Fig. 2d), where both the filament lifetime and extensional relaxation time are over ten-fold longer for trials measured without versus with environmental control (Table 1). The uncertainty in the extracted value of $\lambda _{E}$ for trials outside of the chamber is nearly as large as $\lambda _{E}$ itself, stemming from the drastically different thinning profiles obtained across trials (SI.13). Notably, trials performed in the chamber exhibit highly reproducible thinning (SI.13), where uncertainties in $\lambda _{E}$ are $\le$10% of $\lambda _{E}$. Furthermore, 2D images during thinning inside the chamber follow the same progression for both PEO/NMF and PEO/chloroform (Fig. 2 insets), suggesting that undesirable evaporation during thinning is successfully mitigated by using the chamber.

Interestingly, evaporative effects are substantially more pronounced in chloroform versus DCM (20$\times$ vs. 1.4$\times$ increase in $\lambda _{E}$), despite the fact that DCM has a boiling point over 20 $^{\circ }$C higher than chloroform (Table 1). Chloroform and DCM have nearly identical surface tensions (Table 1), suggesting that these differences are not driven by solvent surface tension. However, the average extensional relaxation time is $\sim$ 30% longer in chloroform versus DCM ($\lambda _{E}=$ $4.4 \pm 0.3$ ms vs. $3.4 \pm 0.2$ ms, respectively), scaling exactly with the solvent viscosity, which is $\sim$ 30% higher in chloroform than in DCM (Table 1). For the semi-dilute, unentangled polymer solutions, $\lambda _{E}$ is predicted to scale as^59,62^:

5 $$\begin{aligned} \lambda _{E}\approx \frac{\eta _{s}b^{3}}{kT}N^{2}\phi ^{(2-3\nu )/(3\nu -1)} \end{aligned}$$

where *kT* is the thermal energy and *b* is the Kuhn length. Given that $\lambda _{E}$ directly scales with $\eta _{s}$, and that $\eta _{sp}$ is approximately equal between the two solutions, Eqs. (4)–(5) suggest that the Flory exponents are similar for PEO in chloroform and in DCM. The observed difference in $\lambda _{E}$ between PEO in chloroform versus DCM is thus most likely attributable to differences in $\eta _{s}$; whereas differences in $\lambda _{E}$ between these two solutions and PEO in water or NMF is likely due to both solvent viscosity and quality. Given the similarity in solvent quality for PEO in chloroform and DCM, we hypothesize that the apparent discrepancy in magnitude of evaporation effects and solvent volatility result because of reduced PEO mobility in chloroform due to higher $\eta _{s}$, to be explored further below.

### Concentration scaling in chloroform

**Figure 3:**
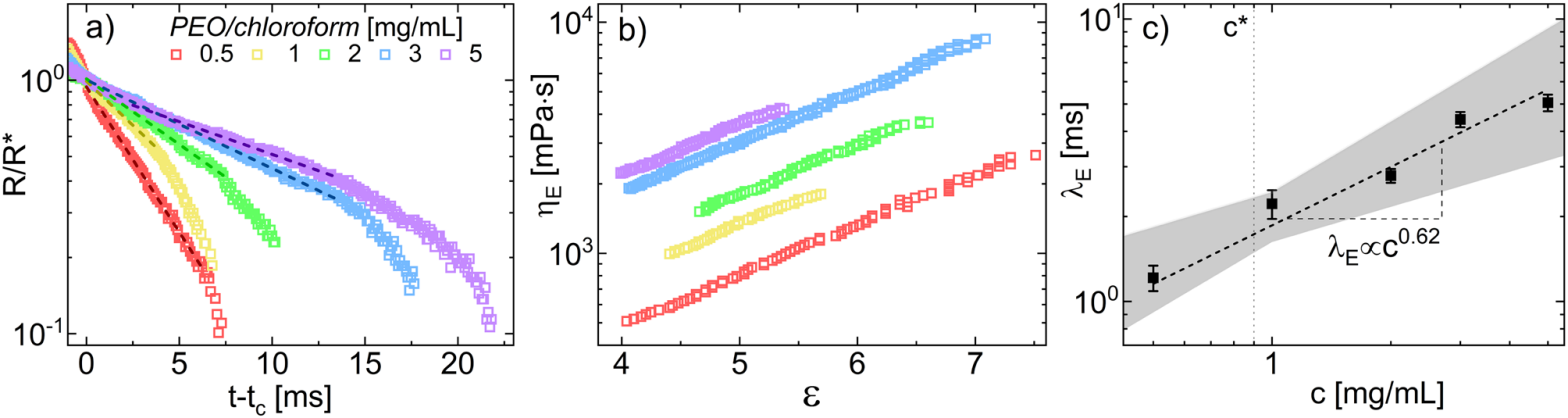
(**a**) Radius evolution, (**b**) extensional viscosities $\eta _{E}$, and (**c**) associated relaxation times $\lambda _{E}$ for semi-dilute PEO/chloroform solutions. Similar to PEO/water solutions^23^, the EC regime lengthens (**a**) and $\eta _{E}$ increases (**b**) with increasing PEO concentration. (**c**) Relaxation times scale with concentration as $\lambda _{E} \propto c^{0.62}$. Error bars are the standard deviation across multiple trials; the shaded region reflects the 95% confidence interval around the fit to average values.

As chloroform solutions display the most drastic evaporation effects, the extensional relaxation times of PEO/chloroform solutions were measured at a number of concentrations in the dilute and semi-dilute regimes to confirm that evaporation is sufficiently mitigated by the chamber. These measurements enable the solvent quality of chloroform to be determined via known scalings between concentration and $\lambda _{E}$ (Eq. 5), providing an additional metric by which to validate the environmental control capabilities of the DoS sample environment. Representative radius evolution plots, extensional viscosities, and extensional relaxation times are shown in Fig. 3.

As $\phi$ scales with the concentration *c*, solvent quality can be measured according to Eq. (5) by fitting the average relaxation times to a power law $\lambda _{E}=Ac^{m}$, where $m=\frac{2-3\nu }{3\nu -1}$ and ranges from 0.31 (good solvent) to 1 (theta solvent). Here, the critical overlap concentration $c^{*}$ marking the nominal onset of the semi-dilute regime was estimated using the intrinsic viscosity $[\eta ]$ both calculated from rheology (SI.8) and from the Mark-Houwink-Sakurada equation, where $c^{*}\approx$ $\frac{1}{[\eta ]}$ (SI.7). As shown in Fig. 3, for PEO in chloroform, $m=0.62$, corresponding to a Flory exponent of $\nu =0.54$. This finding is consistent with better solvation in chloroform than in water, where over a comparable concentration range, water is a theta solvent ($m=1$, $\nu =0.5$)^53^. However, chloroform is still far from the ideal good solvent ($m=0.31$, $\nu =0.588$), which combined with its high vapor pressure, may lead to enhanced evaporation effects and film formation on the free surface of the liquid bridge, causing dramatic differences between extensional relaxation times observed with and without environmental control.

We note that in Fig. 3, *m* is fit including one point in the dilute regime, as $c^{*}$ is an estimate and changes in scaling of $\lambda _{E}$ with concentration do not always align perfectly with $c^{*}$^23,53^. If only the four points above $c^{*}$ are used to determine $\nu$, a similar result is obtained ($\nu =0.55$). Notably, dilute aqueous PEO scales similarly to the semi-dilute PEO/chloroform here, as $\lambda _{E} \propto c^{0.65}$ ($\nu =0.535$). Although the scaling in Eq. (5) is derived from a blob theory for semi-dilute, unentangled polymer solutions^62^, the stretching of polymer chains from extensional flow can lead to screening of excluded volume interactions^53^, potentially increasing the value of *m* (lowering $\nu$) regardless of solvent quality for sufficiently stretched chains. Another consequence of these stretch-induced polymer chain interactions is that polymer solutions considered dilute based on their equilibrium conformation may significantly overlap, such that their relaxation times still scale according to the semi-dilute theory shown in Eq. (5)^23,51,53^. However, regardless of whether the concentration scaling for PEO/chloroform ($\nu =0.54$) is compared to aqueous dilute PEO ($\nu =0.535$) or aqueous semi-dilute PEO ($\nu =0.5$), chloroform is the better solvent.

### Surface effects in free chloroform evaporation

To further explore evaporation and free surface effects, we consider two possibilities: (1) that evaporation occurs homogeneously, where the sample uniformly concentrates with evaporation, or (2) inhomogeneously, where polymer concentrates at the drop surface and forms a viscoelastic film. Both homogeneous and inhomogeneous evaporation would increase the observed relaxation time. However, as chloroform is less volatile than DCM but exhibits more severe evaporation effects, a homogeneous increase in PEO concentration proportional to the solvent evaporation rate is insufficient to fully explain the order-of-magnitude difference in $\lambda _{E}$ for trials in chloroform compared to DCM outside of the chamber. However, formation of a surface film or surface inhomogeneities during evaporation in dilute PEO/chloroform solutions would better explain these observations.

**Figure 4:**
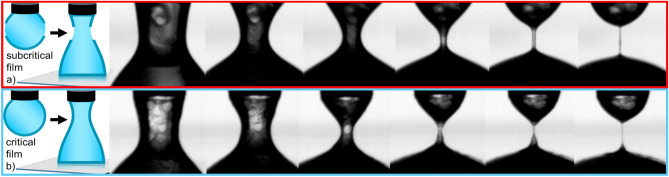
DoS images and schematic of proposed film formation for 3 mg/mL PEO/chloroform (no chamber). (**a**) Trial 1 exhibits subcritical film formation, where a connected film fails to span the entire bridge surface. (**b**) In trial 2, a critical film spans the entire surface, lengthening $t_{b}$ and $\lambda _{E}$ substantially. In both trials, the film has already formed by the time the drop contacts the substrate and begins to thin (frame 1). However, while trial 1 maintains axial symmetry, symmetry is broken in trial 2 and the surface film dominates thinning, explaining the large deviations in $\lambda _{E}$ and breakup time for PEO/chloroform trials performed outside of the chamber.

Images during thinning from two open DoS trials on PEO in chloroform confirm this hypothesis, where a visible surface film forms on the liquid bridge (Fig. 4). We postulate distinct thinning mechanisms for each trial, explaining inconsistencies in the radial decay profiles and large uncertainties in $t_{b}$ and $\lambda _{E}$ for PEO/chloroform measured outside of the chamber (Table 1, SI.13). In Fig. 4a, thinning proceeds fairly uniformly and the filament remains axisymmetric despite the presence of the surface layer. These images suggest a ‘subcritical’ film forms that does not fully cover the droplet surface, leading to $\lambda _{E}$ that are $\sim$ 2–3$\times$ longer than trials taken in the chamber (SI.13). In the final frame, a phenomenon reminiscent of beads-on-string occurs when a portion of the film fractures from the upper bulb and slides down the filament. However, the relatively modest increase in $\lambda _{E}$ and observed axisymmetric thinning suggests that the subcritical film does not dominate the entire thinning process, and that evaporation-driven concentration changes may also increase $\lambda _{E}$.

Conversely, axial symmetry is broken due to the ‘critical’ film which spans the bridge surface in trial two (Fig. 4b). The fully-connected film initially tilts and distorts the filament (frames 2–3); this feature then evolves to form a notch on the filament (frame 4). As the elasticity of the film becomes the primary force resisting thinning, $\lambda _{E}$ is over 30-fold larger for these versus in-chamber trials (SI.13). Eventually, the notch forms a thinner filament in the upper half of the bridge, whereas a thicker region containing more of the surface film remains in the lower half (frame 5). In the final frame, most of the film has drained and a nearly axisymmetric filament is finally observed.

Time lapse videos of pendant drops of 3 mg/mL PEO/chloroform inside and outside of the chamber capture inhomogeneous droplet surface features for open trials, further supporting the proposed film formation (see SI.9). While in-chamber trials appear homogeneous, 2D images for open trials show clear evidence of a film forming and contracting as solvent evaporates (Fig. S10). Notably, changes in the measured surface tension in the open trials are insufficient to diagnose film formation, as $\sigma$ decreases by only 10% over 35 s (Fig. S11). Film formation is evident from the start of the video, suggesting that evaporation begins before the drop fully forms. The wrinkling in the DoS images (Fig. 4) and on the pendant drop surface (Fig. S10a) is consistent with a buckling instability observed in the evaporation of sessile drops^49,50^.

Surface film formation is governed by a Péclet number $Pe=\frac{R_{0}J}{D}$^50^, where $R_{0}$ is the initial drop or bridge size, *J* is the evaporative flux of solvent per unit area, and *D* is the polymer diffusivity in the solvent. Film formation in PEO/chloroform indicates a higher Péclet number than in PEO/DCM, as the evaporative flux of solvent pushing the polymer toward the droplet free surface outpaces polymer diffusion away from the surface. Without environmental control, *J* for dilute solutions is primarily determined by the solvent vapor pressure, where faster drop evaporation times observed in PEO/DCM versus PEO/chloroform (SI.10) suggest that $J_{DCM}>J_{chloroform}$. As the initial drop sizes are equal, PEO diffusion from the surface must then be slower in chloroform than in DCM. Slower diffusion in chloroform is consistent with the reduced PEO mobility in chloroform due to higher $\eta _{s}$ and the higher flow activation energy in chloroform determined from shear rheology (SI.8).

## Discussion

The evaporation-controlled DoS methodology presents the first environment to control evaporation in dripping-onto-substrate extensional rheology measurements of low-viscosity, volatile fluids. Aside from employing an oil bath^33^, suitable only for immiscible samples, or a chamber without a reservoir to generate an enriched solvent atmosphere^42,45^, capillary thinning extensional rheology measurements of volatile systems typically rely on reducing measurement time to reduce evaporation^5,40,41^. Thus the severity of evaporation effects cannot be easily quantified, due to the short timescale on which they occur^45^. While evaporation is exacerbated by the small droplet volume in DoS measurements, comparative measurements of PEO/chloroform using CaBER (SI.18), which requires a larger sample volume and has a smaller surface area-to-volume ratio, still show significant lengthening of the thinning process due to evaporation. Additionally, the step-strain required to form the liquid bridge in CaBER is unsuitable for measuring these low viscosity, low surface tension solutions, as clear oscillations during thinning are observed (Fig. S29). Therefore, surface film formation makes measuring the extensional rheology of polymer solutions in solvents like chloroform nearly impossible without environmental control.

While PEO solutions in both DCM and chloroform exhibited significant evaporation outside the environmental control chamber, chloroform solutions exhibited drastically larger evaporative effects, with an average increase in $\lambda _{E}$ of 2000% for chloroform solutions compared to 40% for DCM solutions. These dramatic differences in evaporation in chloroform solutions are attributed to surface film formation, visually confirmed with additional DoS measurements and from time lapse videos during evaporation (Figs. 4 and S10). Given the lower vapor pressure of chloroform versus DCM, these results suggest that in addition to solvent volatility, evaporation effects and film formation during DoS measurements are a function of solvent viscosity and chain mobility.

The speed of film formation is related to a critical Péclet number at which a film forms $Pe=\frac{R_{0}J}{D}$^50^. This critical Péclet number can also be defined in terms of the initial solute concentration $\phi _{0}$ and the critical concentration for film formation $\phi _{g}$ as $\frac{\phi _{g}-\phi _{0}}{1-(\phi _{0})\phi _{0}}$^50^. Given that drops of each PEO solution have the same initial size and concentration, and that evaporative flux is higher for DCM solutions than for chloroform solutions (SI.10), film formation in chloroform solutions indicates slower diffusion of PEO, or a lower critical concentration of film formation. While the specific viscosities for PEO in chloroform and DCM are similar (Table 1), the higher solvent viscosity of chloroform and the higher flow activation energy for PEO in chloroform (SI.8) indicate reduced mobility for PEO in chloroform versus DCM.

While film formation due to solvent evaporation is necessary in fiber spinning processes, the precise mechanism by which polymer solutions form these films is still an open question. The films have been considered as gel-like phases^50^, the formation of which is likely influenced by extension-induced orientation^47^. Extensional alignment and increased concentration near the surface can lead polymer solutions to form an aligned surface layer,^63^ a core-shell morphology with a well-defined outer phase^64,65^, or a supramolecular structure comprised of several nanofibers^66^. In each case, rapid evaporation at the surface of the liquid leads to higher surface concentration^67^ and a highly elastic surface layer in spun fibers^47,66^; as such, a similar process is likely occurring in freely evaporating PEO/chloroform.

While evaporation may not be a major concern for ambient, short timescale DoS measurements in solvents less volatile than water, the impact of evaporation on these measurements in volatile solvents cannot be predicted precisely either without knowing properties like the solvent quality, polymer diffusivity, and evaporative flux, or without explicitly controlling for evaporation phenomena. In addition to enabling measurement of volatile organic solutions, evaporative control is likely desirable for measuring solutions in relatively non-volatile solvents at elevated temperatures or for extended times, especially if these solvents do not dissolve their solutes well. Therefore, the evaporation-controlled DoS technique provides an important new tool for measuring the extensional rheology of low-viscosity volatile systems, and for detecting and quantifying evaporation effects on the measured rheological parameters.

## Methods

### Materials

Polyethylene oxide ($M_{W}=10^{6}$ g/mol, DP = 22,700) was purchased from Beantown Chemical. ACS-grade chloroform (Fisher Scientific), ACS-grade dichloromethane (DCM, Macron Chemical), 99% N-methylformamide (NMF, Sigma-Aldrich), and high performance liquid chromotography (HPLC)-grade water (Fisher Scientific) were used as-received. Solutions were prepared by dissolving powder in warm solvent, and shaking at 4 $^{\circ }$C for 24 h. Low concentration PEO/chloroform solutions were prepared by dilution from a 3 mg/mL stock solution.

### Dripping-onto-substrate (DoS) measurements

The DoS instrument used in this work consists of a 2600 lumen light source, a high-speed camera capable of recording 38,000 frames per second, a syringe pump, and a syringe with a nozzle of outer radius $R_{0}$. This syringe is mounted directly above the substrate, rather than using tubing as in other DoS instruments^52^ to minimize pre-deformation^38^, and eliminate leaching of compounds from the tubing due to incompatibility with solvents used. To form the liquid bridge, the syringe pump is used to extrude a drop nearly until the point of contact with the substrate at a very low flow rate, typically below 0.1 mL/h. To minimize fluid oscillations resulting from pump vibrations in this “direct mount” approach, the pump is subsequently turned off, and the pendant drop and substrate are brought into contact to form the liquid bridge^38^.

“Open” configuration trials were performed without the environmental control chamber shown in Fig. 1. The approximate aspect ratio $\frac{h}{2R_{0}}$ was adjusted to $\sim$ 1.4, with $R_{0}$ fixed to 0.82 mm. While results for aqueous polymer solutions are nearly independent of syringe pump flow rate below 0.5 mL/h^23^, trials conducted using the ‘direct mount’ approach (Fig. 1) with the pump on exhibited inertially-driven oscillations on the liquid bridge surface (SI.4)^30^. To remedy this problem, the pump is turned off prior to drop contact with the substrate and the stage is slowly moved to contact the drop, as previously described^38^.

For all “closed” DoS measurements in the chamber, the reservoir was filled with 100 mL solvent. Before sealing the chamber, the aspect ratio ($\frac{h}{2R_{0}}$) between the needle and substrate is adjusted using the camera. The aspect ratio was $\sim$ 1.4 for all DoS trials, with $R_{0}$ fixed to 0.82 mm. The chamber atmosphere was allowed to saturate with solvent vapor for 45 min before drop extrusion. This equilibration period was standardized across solvents for comparison purposes; however, long equilibration periods generally reduced evaporation rates (SI.10), enabling evaporation effects to be tuned. Evaporation rates were quantified via time-lapse videos of evaporating pendant drops, taken after the equilibration period (SI.10). The syringe was then filled with sample and lowered to pierce the septum. As the stage could not be moved to meet the drop while in the chamber, the needle was instead slowly depressed to initiate substrate contact. This mode of operation was least prone to introducing surface oscillations of the liquid bridge^30^, likely due to mechanical dampening from the septum.

High-speed videos of the filament radial decay were recorded for all trials (open and closed configurations) at 11,800 fps, with a spatial resolution of 288 by 326 pixels. Individual frames were thresholded in ImageJ and analyzed in Matlab to extract values of the minimum radius in time; see SI.1 for more details. Extracted radii were normalized to the radius at the transition between IC and EC regimes, $R^{*}$. Despite the lower surface tension in the selected volatile solvents (Table 1), contributions from gravitational sagging are unimportant in the analyzed thinning processes; see SI.5 for Bond number calculations.

### Solvent quality analysis

Solvent quality was first estimated using values of the relative energy density (RED) calculated from Hansen solubility parameters^55^, where smaller values correspond to better solvents, and values larger than unity indicate poor solvent quality. Solvents were thus selected based on an RED value of $\le$ 1 for the solvent and PEO. To determine the onset of the semi-dilute regime, the critical overlap concentration $c^{*}$ was estimated for PEO in water ($c^{*}=1.7$ mg/mL) and chloroform ($c^{*}=1.0$ mg/mL) were estimated using intrinsic viscosity $[\eta ]$ from the Mark-Houwink-Sakurada equation as $c^{*}\approx \frac{1}{[\eta ]}=\frac{1}{{KM_{W}}^{a}}$. The values of *K* and *a* for PEO in chloroform were estimated based on published values of intrinsic viscosity of PEO in chloroform (see SI.7)^68–70^.

### Shear rheology

As both $c^{*}$ and $[\eta ]$ are sensitive to the PEO molecular weight and molecular weight distribution, shear rheology was performed to support calculations and to determine the specific viscosity, $\eta _{sp}=\frac{\eta _{0}-\eta _{s}}{\eta _{s}}$, for each sample; see SI.8. Briefly, steady shear rheology was performed using an Anton Paar MCR 302 stress-controlled rheometer with a 26.7 mm double-gap geometry and solvent trap. This geometry was selected to maximize surface area and thus the torque signal, to enable accurate measurements of low viscosity solutions. As these solutions are low viscosity and are nearly-Newtonian across a range of shear rates, solutions were measured at several shear rates to obtain improved torque signal. The zero-shear viscosity, $\eta _{0}$, was calculated by averaging the shear viscosity across the Newtonian shear rates, and in most cases, across two trials. Reported error bars are the standard deviation of the average $\eta _{0}$ values. As highly volatile solutions could not be measured at $25\,^{\circ }$C to evaporation effects, reported values were extrapolated from low temperature data based on an Arrhenius-like scaling with temperature (Eq. S3, see SI.8.1.1 for details). Subsequent analysis of shear rheology was in excellent agreement with calculations, giving $c^{*}=1.7$ mg/mL for PEO/water, and $c^{*}=0.9$ mg/mL for PEO/chloroform.

### Parameter fitting and statistics

Extensional relaxation times were determined by fitting the elasto-capillary regime data to Eq. (3) following a binning procedure; see SI.1 for details. Uncertainty is reported as the standard deviation of $\lambda _{E}$ between trials, which was much larger than the fit uncertainties. Surface tension values were extracted from the pendant drop shape using an ImageJ plugin^71^.

To assess if statistically significant differences resulted between freely evaporating and evaporation-controlled DoS measurements, two-sample, one-tailed Student’s *t*-tests (assuming unequal variances) were performed, where values of $\lambda _{E}$ were compared in the open and closed configuration. The null hypothesis was that the mean $\lambda _{E}$ values were equal. As evaporation effects increase the timescales for thinning, the alternative hypothesis was that $\lambda _{open}>\lambda _{closed}$. Differences were determined to be statistically significant at the 95% confidence level based on *p*-values (see SI.3).

## Supplementary Information

Supplementary Information.
