# Supplementary material for: Evaporation-controlled dripping-onto-substrate (DoS) extensional rheology of viscoelastic polymer solutions
Source: Sci Rep. 2022 Mar 18;12:4697. doi: 10.1038/s41598-022-08448-x (PMC8933544; doi:10.1038/s41598-022-08448-x)
Supplement: Supplementary file 1 — Supplementary Information. [file 41598_2022_8448_MOESM1_ESM.pdf]

Supporting Information: Evaporation-controlled dripping-onto-substrate (DoS) extensional rheology of viscoelastic polymer solutions

Benjamin P. Robertson and Michelle A. Calabrese*

Department of Chemical Engineering and Materials Science
University of Minnesota, Minneapolis, Minnesota, 55455

*Email: mcalab@umn.edu

Contents

SI.1 Image processing and fitting of high speed videos S2
SI.2 Environmental control chamber S2
SI.3 Additional parameter values S3
SI.4 Droplet surface oscillations S3
SI.5 Calculated Bond numbers S3
SI.6 Calculation of relative energy densities S4
SI.7 Estimation of concentration regimes for PEO in water and chloroform using prior literature S4
SI.8 Shear rheology: estimates of concentration regimes and dimensionless numbers S5
SI.8.1 Methodology and analysis S6
SI.8.1.1 Temperature-controlled studies of PEO in water and chloroform S6
SI.8.1.2 Analysis S6
SI.8.2 3 mg/mL PEO in water and chloroform S7
SI.8.3 Concentration series: PEO/water S7
SI.8.4 PEO in chloroform S8
SI.8.5 PEO in DCM and NMF S10
SI.9 Time lapse videos and surface tension of 3 mg/mL PEO in chloroform S12
SI.10 Evaporation studies on pendant drops S13
SI.11 Evaporation and extension of chloroform solutions after 2 hours equilibration S15
SI.12 Estimates of extensional relaxation time for homogeneous concentration S17
SI.13 Individual trials and EC fits S18
SI.14 Extension rates and extensional viscosities S20
SI.15 Individual trials and IC fits S22
SI.16 Terminal visco-elasto-capillary (TVEC) fits S25
SI.17 Volume comparisons between methods S26
SI.18 Capillary Breakup Extensional Rheometry (CaBER) S27
SI.19 Variables and abbreviations S28

SI.1 Image processing and fitting of high speed videos

Raw videos were cropped to include only the region between the needle and the substrate, then resized by a factor of 10, interpolating with a bicubic algorithm for subpixel resolution. These videos were then thresholded in ImageJ at the last peak in intensity using the 16-bit values from the raw video, (between 0 and 65535), to produce binarized videos. Binarized videos were converted in Matlab to 2D discrete data of the function $r(z,t)$, which was trimmed to remove points before contact of the substrate and after breakup.

The fitting region for the elasto-capillary (EC) regime was determined by binning each trial and fitting to Equation 3 within each bin. The EC regime was determined to consist of consecutive bins in which the value of λ_E was statistically equal and did not trend in time. This criterion corresponded visually with the onset of the linear region of the minimum radius profile as well as the final decay indicating the failure of edge detection. This fitting region was then fit to Equation 3 extract a single value of λ_E, and the radius of the liquid bridge was normalized by the radius at the onset of the EC regime R^*. Uncertainties from each fit were negligible in comparison to the variation between trials, so uncertainties for average values of λ_E for each sample and evaporation condition were calculated as the standard deviation of the fit values for each trial.

Based on an 288 by 326 pixel image of a needle with outer diameter of 1.63 mm, the theoretical maximum spatial resolution is 5.7 μm, assuming the needle takes up the entirety of the image. In reality, the chamber wall placement prevents the camera from being placed too close, and the thresholding process is unable to capture the end of thinning, due to the contrast of the fluid decreasing as the filament thins. The highest spatial resolution achieved in the measurements taken in the chamber is 25 μm, or R/R_0 of 0.015. However, the procedure of resizing to achieve subpixel resolution allows differences in filament size less than this maximum spatial resolution to be captured, down to 5 μm, but the minimum size of the filament that can be captured is still limited by the size of the pixel. This means, for example, that the difference between radii of 30 pixels and 30.5 pixels is still meaningful, but the difference between 0.5 and 1 pixels is not.

SI.2 Environmental control chamber

An environmental control chamber was added to a direct-mount DoS instrument created by Lauser[1] in order to produce an atmosphere enriched in solvent vapor.

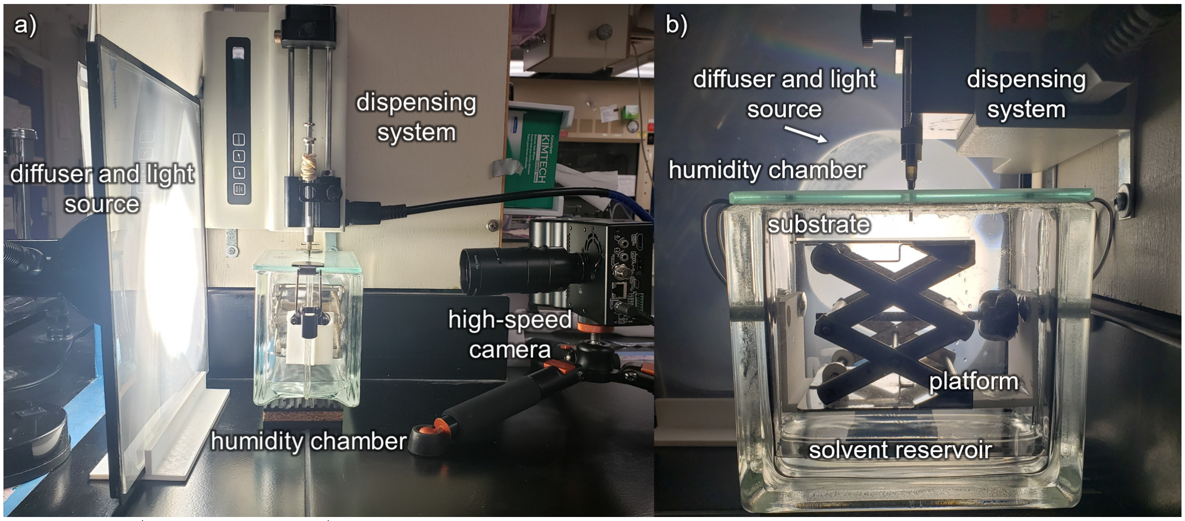

Figure S1: a) Front and b) side views of evaporation-controlled DoS instrument in operation, adapted from the design by Lauser[1] based on work by Dinic and Sharma.[2]

SI.3 Additional parameter values

Table S1: Reported solvent surface tensions[3,4] and p-values, calculated by one-tail t-test, to indicate whether or not there are statistically significant differences in relaxation times obtained with the chamber open and closed for PEO in various solvents. P-values indicate that water and NMF exhibit no statistically significant difference in λ_E due to evaporation, whereas chloroform and DCM exhibit significantly longer values of λ_E.

solvent	solvent σ [mN/m][3,4]	p-value
water	72	0.16
NMF	38	0.46
DCM	27	0.04
chloroform	28	0.03

SI.4 Droplet surface oscillations

In organic solvents with low surface tension and low viscosity, inertial effects upon contact between drop and substrate can cause oscillations visible on the free surface of the liquid bridge. These oscillations, seen most clearly in the fourth frame of Figure S2 at 9.3 ms, are evidence of a small pressure wave travelling vertically through the thinning liquid bridge. Similar effects have also been observed in CaBER[5], due to the inertia of plate separation, but the surface tension is often high enough for aqueous samples measured via DoS that these oscillations die down extremely quickly. Whereas the second frame (3.1 ms) would be considered the “start point” due to the approximately cylindrical shape of the liquid bridge, R in the third frame is larger than in the second frame. This behavior appears as a 1D “bounce,” contrary to the predictions of Equation 2, preventing the use of trials exhibiting oscillations for analysis.

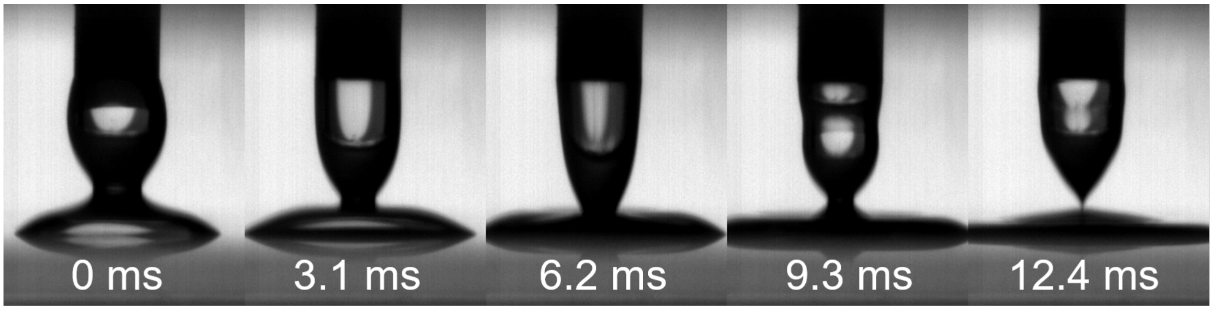

Figure S2: A pressure wave in a thinning liquid bridge of acetone leads to the expansion of the neck at 6.2 ms and a non-monotonic profile of $R(t)$

SI.5 Calculated Bond numbers

The dimensionless Bond number quantifies the impact of gravitational versus surface tension forces, given by:

$$Bo = \frac{\rho g R^2}{\sigma} \tag{S1}$$

where $\Delta\rho$ is the difference in density between the air and solution (approximated as the solvent density, g is gravity, and R is the characteristic radius. Here, we calculate an initial Bo at the start of thinning using $R = R_0$, and a local Bond number at the transition radius between IC and EC thinning ($R = R^*$), shown in Table S2. Another local Bond number is calculated based on a critical transition radius R_c determined at an intrinsic Deborah number ($De_0 = \lambda_E/(\rho R_c^3/\sigma)^{1/2}$) of

unity; here the denominator is simply t_R using R_c instead of R_0. Note that experimentally, the EC transition occurs at radii smaller than R_c predicted by De_0.

As seen in Table S2, all Bo are far below unity, indicating that surface tension forces dominate gravitational forces. Clasen[6] showed that below a local Bond number of approximately 0.2, contributions from gravitational sagging do not substantially impact the observed thinning processes. Both methods of calculating the local Bo for the onset of the EC transition yield values below 0.2 for PEO in all solvents. As such, gravitational forces are not important contributions to the thinning processes at these radii, and can be ignored in the analysis of EC thinning in each solvent examined.

Table S2: Bond numbers calculated near the start of thinning ($R = R_0$) and at the transition between IC and EC thinning ($R = R^*$). R^*/R_0, the normalized radius at the EC transition onset, is also given; uncertainty is the standard deviation based on multiple trials.

solvent	Bo ($R = R_0$)	Bo ($R = R^*$)	R^*/R_0
Water	0.106	$1.93 \cdot 10^{-3}$	0.14 ± 0.01
NMF	0.180	$1.40 \cdot 10^{-3}$	0.09 ± 0.02
DCM	0.350	$6.87 \cdot 10^{-3}$	0.14 ± 0.01
chloroform	0.339	$7.21 \cdot 10^{-3}$	0.15 ± 0.03

SI.6 Calculation of relative energy densities

Relative energy density (RED) can be used to estimate solvent quality for polymers, using three solubility parameters for dispersion (δ_D), polar (δ_P), and hydrogen bonding (δ_H) interactions. Placing these three parameters in 3-dimensional space, the distance between a polymer and a solvent can be calculated and compared to an interaction radius R_o for that polymer according to Equation S2:[7]

$$RED = \frac{R_a}{R_o} = \sqrt{4(\delta_{D2} - \delta_{D1})^2 + (\delta_{P2} - \delta_{P1})^2 + (\delta_{H2} - \delta_{H1})^2} \tag{S2}$$

For a value of RED ≤ 1, the solvent is predicted to dissolve the polymer, with lower values suggesting better solvents. According to the RED values in Table S3, water, NMF, DCM, and chloroform should all be good solvents for PEO, with all the organic solvents being better than water for PEO.

Table S3: Hansen solubility parameters for dispersion (δ_D), polar (δ_P), and hydrogen bonding (δ_H) interactions are listed from Hansen[7], and used to calculate relative energy density (RED) according to Equation S2.

	δ_D [MPa$^{1/2}$]	δ_P [MPa$^{1/2}$]	δ_H [MPa$^{1/2}$]	R_o [MPa$^{1/2}$]
PEO	21.5	10.9	13.1	15.9
Solvent	-	-	-	RED
Water	15.1	20.4	16.5	1.03
NMF	17.4	18.8	15.9	0.74
DCM	18.2	6.3	6.1	0.67
Chloroform	17.8	3.1	5.7	0.82

SI.7 Estimation of concentration regimes for PEO in water and chloroform using prior literature

The critical overlap concentration c^*, which defines the onset of the semi-dilute regime in which polymer coils begin to interact, was estimated using intrinsic viscosity $[\eta]$ from the Mark-Houwink-

Sakurada equation as $c^* \approx \frac{1}{[\eta]} = \frac{1}{K M_W{}^a}$. For PEO in water, published experimental values of $K = 6.103 \cdot 10^{-3}$ mL/g and $a = 0.83$ at 25 °C were used to calculate an intrinsic viscosity of $[\eta] = 582.83$ mL/g.[8] For PEO in chloroform, published experimental values of $[\eta]$ were used to estimate parameters of $K = 1.807 \cdot 10^{-2}$ mL/g and $a = 0.79$ at 25 °C (Table S4).[8–10] Whereas the critical concentration of entanglement, c_E, defining the upper bound of the semi-dilute concentration regime, was not measured for PEO samples in chloroform, c_E for 10^6 g/mol PEO in water has been reported as 49 mg/mL,[11] an order of magnitude above the highest concentration chloroform sample in this work.

Table S4: Literature values of intrinsic viscosity $[\eta]$ for PEO of various molecular weights M_W, and the extrapolated $[\eta]$ for the PEO used in this study, where $M_W = 10^6$ g/mol.

M_W [g/mol]	$[\eta]$ [mL/g]
$1{\cdot}10^5$	173.2[9]
$3{\cdot}10^5$	339[10]
$6.47{\cdot}10^5$	778[8]
$1{\cdot}10^6$	1011 (extrapolated)

Literature and extrapolated values of $[\eta]$ are plotted with a linear fit in Figure S3.[8–10] Based on the extrapolated value of $[\eta] = 1011$ mL/g, the critical overlap concentration can be estimated for PEO in chloroform as $c^* \approx 1.0$ mg/mL, and for PEO in water as $c^* \approx 1.7$ mg/mL.

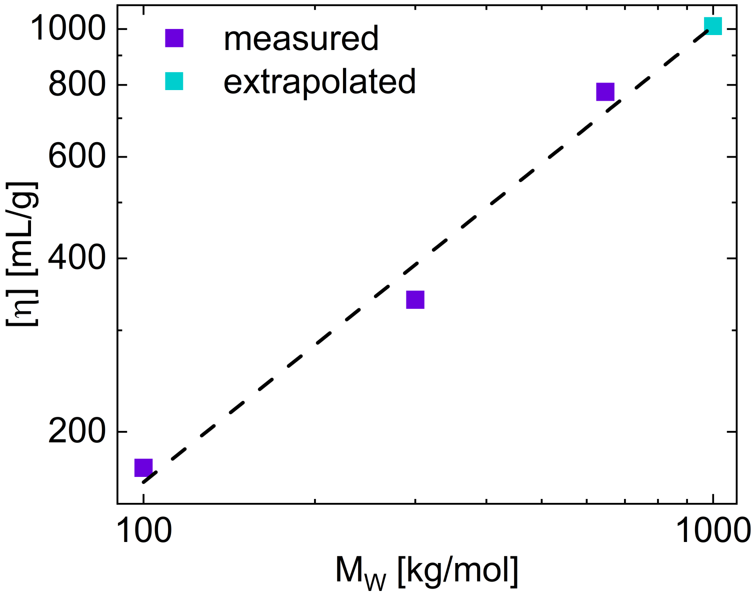

Figure S3: Fitting the Mark-Houwink-Sakurada equation to experimentally measured values,[8–10] $[\eta]$ constants are estimated for PEO in chloroform as $K = 1.807 \cdot 10^{-2}$ mL/g and $a = 0.79$, allowing the estimation of intrinsic viscosity for a given M_W as $[\eta] = 1.807 \cdot 10^{-2} M_W^{0.79}$, so $[\eta]$ for 10^6 g/mol PEO in chloroform can be estimated as 1011 mL/g.

SI.8 Shear rheology: estimates of concentration regimes and dimensionless numbers

As both c^* and $[\eta]$ are sensitive to the PEO molecular weight and molecular weight distribution, shear rheology experiments were performed on a concentration series of PEO in water and in chlo-

roform. Subsequent analyses were then used to confirm the estimated c^* values from SI.7; the estimates using literature data were in excellent agreement with rheology.

SI.8.1 Methodology and analysis

SI.8.1.1 Temperature-controlled studies of PEO in water and chloroform

As shown in Figure S12 below, evaporation of DCM and chloroform at ambient conditions is substantial within less than thirty seconds. Given the longer timescale required to perform shear rheology measurements, PEO in chloroform required measurement at colder temperatures, and the behavior at 25 °C was extrapolated from these trends and subsequently compared to the estimates in SI.7 above. The temperature-dependence of the viscosity of polymer solutions often follows an Arrhenius-like dependence, given by:[12]

$$\eta = Ae^{E_a/RT} \tag{S3}$$

where A is a constant, E_a is the flow activation energy, R is the gas constant, and T is the absolute temperature. Here, E_a is expected to be higher in poorer solvents, in high viscosity solvents, and when the chain flexibility is lower.[13,14] Note that in good solvents, the viscosity can also follow a linear dependence; however, Eqn. S3 was chosen to analyze all data for consistency across sample concentrations.

To validate this approach, the shear viscosity of PEO in water (minimal evaporation) was measured across wide range of temperatures and shear rates. The resulting η_0 were then fit with Eqn. S3 to confirm that the data was well-described by this model. The same approach was then taken for PEO in chloroform, starting at temperatures of 1 °C and 2.5 °C. To determine if significant evaporation occurred at temperatures above 2.5 °C, the data from these temperatures was used a baseline. Following higher temperature measurements, measurements were repeated at 1 °C or 2.5 °C; if the same response was obtained, the sample was determined to be unimpacted by evaporation. Using this approach, no PEO/chloroform samples were impacted by evaporation up to 7.5 °C. At 10 °C, minimal evaporation was observed if measurements were taken quickly; however, some PEO/chloroform samples were impacted by evaporation at 10 °C. As confirmed by the onset of evaporation around 10 °C, accurate shear rheology measurements could not be performed for PEO in volatile solvents at 25 °C, even with a solvent trap. After fitting the data to Eqn. S3, the zero-shear viscosity of each chloroform sample was extrapolated at 25 °C.

SI.8.1.2 Analysis

The intrinsic viscosity $[\eta]$ and c^* can be determined via rheology. On a log-log scale, the PEO zero-shear viscosity vs. concentration, c, will be linear in the dilute regime. The deviation from linearity indicates $c*$, which can also be estimated as $[\eta]^{-1}$. To determine $[\eta]$, the specific viscosity, η_{sp}, and reduced viscosity, η_{red}, were determined for each sample concentration and temperature based on the temperature-dependent η_0 and temperature-dependent solvent viscosity, η_s:

$$\eta_{sp} = \frac{\eta_0 - \eta_s}{\eta_s} \tag{S4}$$

$$\eta_{red} = \frac{\eta_{sp}}{c} \tag{S5}$$

The intrinsic viscosity $[\eta]$ was determined for PEO in water or chloroform as a function of temperature, by fitting the Huggins Equation[15] to the dilute solution data for each temperature:

$$\eta_{red} = [\eta] + k_H[\eta]^2c \tag{S6}$$

where k_H is a constant. Note that because solvent quality is temperature-dependent, both $[\eta]$ and c^* are also temperature-dependent.

SI.8.2 3 mg/mL PEO in water and chloroform

To validate Eqn. S3 for PEO/water and PEO/chloroform, shear rheology measurements were performed on 3 mg/mL PEO in water from 10 to 25 °C and on PEO/chloroform from 1 to 10 °C (Figure S4). Above 10 °C, the chloroform data exhibited significant effects from evaporation. The higher viscosities of these semi-dilute solutions enabled a strong signal across a range of shear rates; thus measurements were only performed up to shear rates of $\dot{\gamma}$=30 s^{-1}. While these solutions are semi-dilute, the viscosity displays only very mild shear thinning with increasing shear rate; more significant shear thinning is expected for PEO/water at higher shear rates.[16] The viscosity for PEO/chloroform is roughly 7% larger than that for PEO/water at equal temperatures, as expected based on the better solvency of chloroform for PEO.

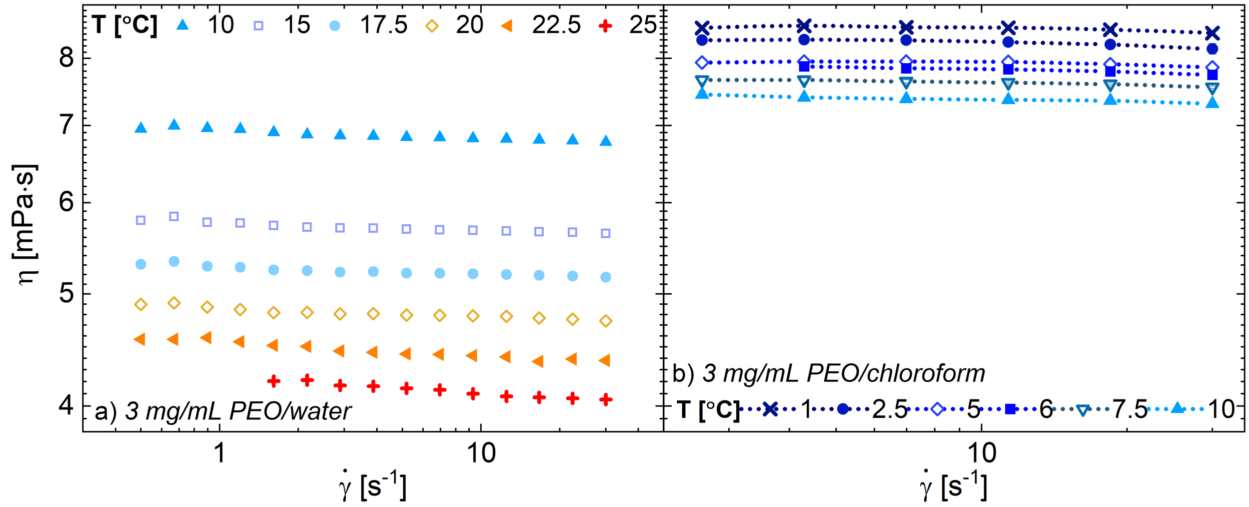

Figure S4: Viscosity as a function of shear rate for 3 mg/mL PEO (1000 kDa) in a) water and b) chloroform as a function of temperature. The solutions display very mild shear thinning at higher shear rates. In chloroform samples, fewer shear rates are measured to minimize evaporation effects; dotted lines are for visual aid only.

The zero-shear viscosities for PEO in both chloroform and water follow an Arrhenius-like dependence on temperature (Eqn. S3, Figure S5). Importantly, when the slope of the line in Figure S5a is calculated using the three or four lowest temperatures for PEO/water, η_0 at 25 °C can be predicted from extrapolation within 3% accuracy, giving confidence in this method for determining η_0 for PEO/chloroform. The slope of the lines in Figure S5a and Figure S5c correspond to E_a/R, giving activation energies of $E_a = 23.4$ kJ/mol and 10.2 kJ/mol for PEO in water and chloroform, respectively. Note that E_a depends on both viscosity and chain mobility, where larger values of E_a correspond to both higher viscosities and lower chain mobilities. That E_a for PEO in water is more than double that of PEO in chloroform further supports the better solvency of chloroform for PEO. Additionally, while the data for PEO/water as a function of temperature can only be described by Eqn. S3, the PEO/chloroform data can also be described by a linear relationship, as has been observed in good solvents.[17]

SI.8.3 Concentration series: PEO/water

Shear rheology measurements on PEO in water were performed at the following concentrations (mg/mL) at 25 °C, following a serial dilution: 3, 2.25, 1.69, 1.27, 0.95, 0.71 and 0.53. In the two most concentrated solutions, a very mild shear thinning is observed at high shear rates ($\dot{\gamma} > 30$ s^{-1}), consistent with semi-dilute solution behavior. For $c \leq 1.27$ mg/mL, solutions exhibit Newtonian behavior over the measured range (Figure S6a). The zero-shear viscosity begins deviating from a linear scaling (on a log-log scale) near the 1.69 mg/mL solution, indicating proximity to c^* (Figure

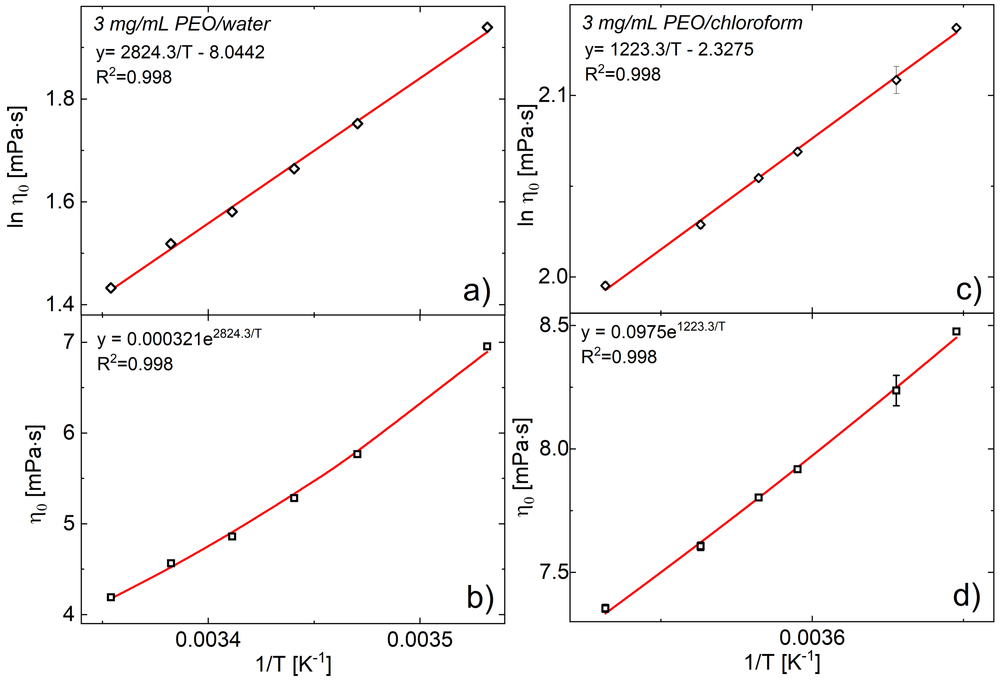

Figure S5: Zero-shear viscosity, η_0, for 3 mg/mL PEO (1000 kDa) in a,b) water and c,d) chloroform as a function of temperature. Both solutions follow an Arrhenius-like behavior (Eqn. S3), shown in a) and c) on a natural log scale (linear fit) and in b) and d) on a linear scale (exponential fit). When the lowest 3 or 4 temperatures for PEO/water are used to extrapolate $\eta_0(25)$, the estimate is within 3% of the true value.

S6b). This solution showed a decrease in η at the highest $\dot{\gamma}$; however, the decrease in η was on the order of the measurement uncertainty. When Eqn. S6 is fit to η_{red} using only the four lowest concentration samples, $[\eta]$ = 564.7 mL/g and c^* = 1.77 mg/mL. This fit indicates that c = 1.69 mg/mL should be included in the fit; when this point is included, $[\eta]$ = 605.8 mL/g and c^* = 1.65 mg/mL– suggesting that this concentration is above c^*. Both fits are thus shown in Figure S6c, and we take the average of both methods to determine $[\eta]$ = 585.3 mL/g and c^* = 1.71 mg/mL, in near-perfect agreement with calculations in SI.7.

SI.8.4 PEO in chloroform

Similar to results for PEO/water, shear rheology demonstrated that PEO/chloroform solutions were nearly Newtonian in the dilute regime, and displayed very mild shear thinning at high shear rates in the semi-dilute regime. Individual trials are not shown except for 3 mg/mL (Figure S4b) given that measurements were performed for five concentrations (0.25, 0.5, 0.75, 1.0 and 3 mg/mL) and up to five temperatures (1, 2.5, 5, 7.5 and 10 °C); however, the temperature-dependence for η_0 and η_{red} for each concentration is summarized in Figure S7 ($c \leq 1$ mg/mL) and Figure S5 (3 mg/mL).

Further supporting this determination of c^*, the slope of the lines in Figure S7a are independent PEO concentration (within statistical certainty) in the region near and below c^*, whereas the slope and thus flow activation energy are statistically larger for c = 3 mg/mL PEO/chloroform (Table S5). In low concentration polymer solutions, E_a has been shown to be independent of concentration in dilute conditions; with further increases in concentration, E_a subsequently increases.[18]

Using the trends from Figure S7, η_0 and η_{red} were estimated for each concentration at 25 °C (Figure S8a,b). Both methods of estimation suggested that c^* was near, but slightly less than the 0.99 mg/mL estimate from SI.7. A linear fit to the reduced viscosity estimate gives an intercept of $[\eta]$ = 1107.2 mL/g, corresponding to c^* = 0.903 g/mL. As each solution was measured at vari-

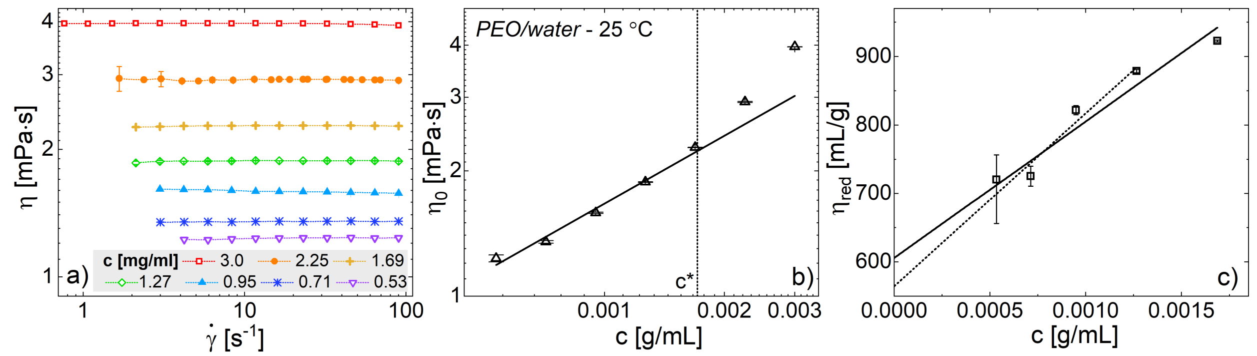

Figure S6: Shear viscosity of 1000 kDa PEO/water at various concentrations. a) Viscosity as a function of shear rate. Below c^*, no shear thinning is observed with increasing shear rate; mild shear thinning is observed at high rates for more concentrated solutions. b) Zero-shear viscosity as a function of concentration, where the deviation from linearity corresponds with $c^* = 1.71$ mg/mL PEO/water. c) A linear fit to the data below c^* gives a y-intercept corresponding to $[\eta]$. Two fits are shown, one with and one without $c = 1.69$ mg/mL.

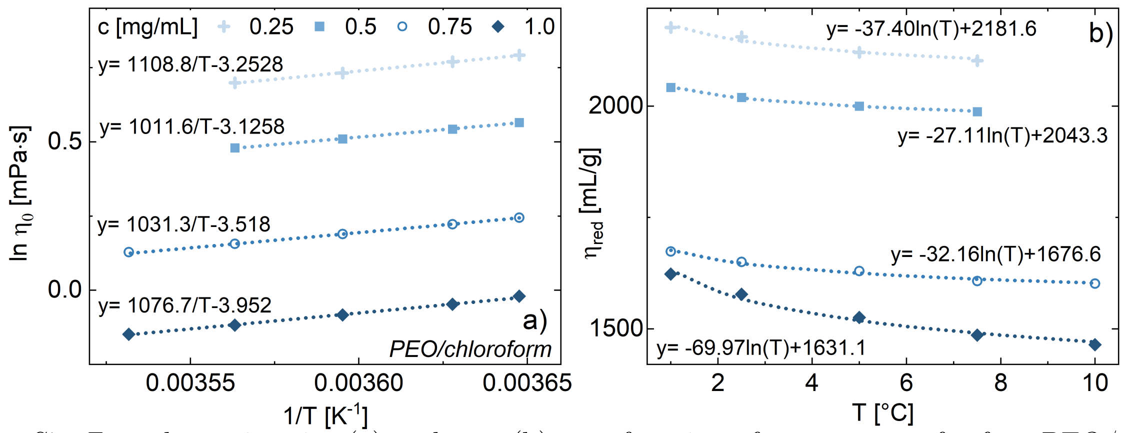

Figure S7: Zero-shear viscosity (a) and η_{red} (b) as a function of temperature for four PEO/ chloroform concentrations. For all linear fits (a), $R^2 \geq 0.997$, and for natural log fits (b), $R^2 \geq 0.97$, indicating excellent fit quality for both temperature trends.

ous temperatures, the intrinsic viscosity at each temperature was also calculated (Figure S8c); $[\eta]$ decreases with increasing temperature as the chloroform solvent quality worsens. To check the estimates of $[\eta]$ and c^* shown in Figure S8a,b, the $[\eta]$ vs. T data was also fit and used to extrapolate a value for $[\eta]$ at 25 °C. These calculations yielded $[\eta]$ = 1105.1 mL/g (c^* = 0.905 g/mL), in excellent agreement with the other estimates.

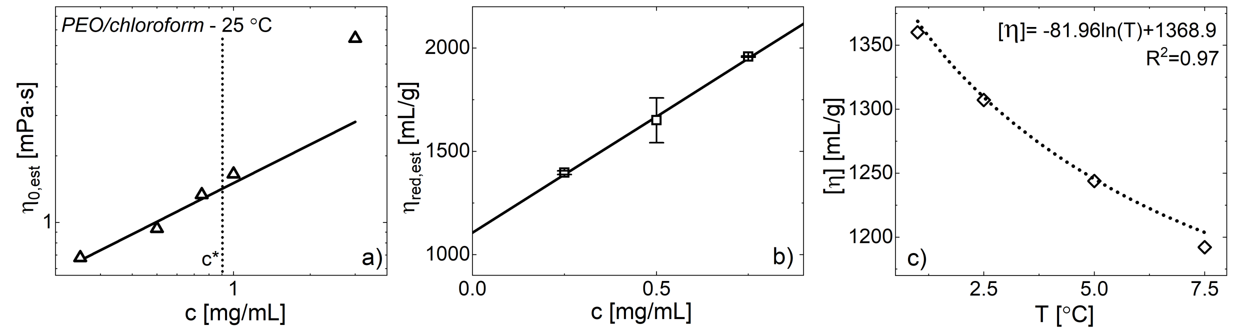

Figure S8: Estimated η_0 (a) and η_{red} (b) for PEO/chloroform, suggesting $c^* \approx 0.9$ mg/mL at 25 °C. c) $[\eta]$ at low T was fit to extrapolate $[\eta]$ at 25 °C, also giving $c^* \approx 0.9$ mg/mL.

Table S5: Parameters extracted from shear rheology temperature series for PEO/solvent combinations. The slope of the $ln(\eta_0)$ vs. $1/T$ curve, associated 95% confidence interval, and extracted E_a are given for samples in which a temperature series was performed. Uncertainty in E_a is estimated based on the reported confidence interval around the slope. Reduced viscosity, η_{red}, is measured for water and NMF samples at 25 °C and extrapolated to 25 °C for DCM and chloroform samples; Oh calculations use $\sigma_{solution}$ at 3 mg/mL and $\sigma_{solvent}$ for lower concentrations. Note that due to solubility and evaporation issues, DCM estimates are based on only three points (5, 7.5 and 10 °C).

**c [mg/mL]**	η_0 **[mPa·s]**	η_{red}	Oh	**slope**	**95% CI**	E_a **[kJ/mol]**
PEO/chloroform						
0.25	0.71	1.3	0.004	1077	1007–1147	9.0 ± 0.6
0.50	0.94	1.7	0.005	1031	965–1097	8.6 ± 0.5
0.75	1.3	2.4	0.007	1012	902–1121	8.4 ± 0.9
1.0	1.6	3.0	0.009	1109	1009–1208	9.2 ± 0.8
3.0	5.9	10.9	0.031	1223	1152–1295	10.2 ± 0.6
PEO/DCM						
3.0	4.2	10.1	0.025	810	784–837	6.7 ± 0.2
PEO/water						
3.0	4.1	4.7	0.018	2824	2636–3012	23.4 ± 1.6
PEO/NMF						
3.0	8.2	4.9	0.047	1909	1858 – 1960	15.9 ±0.4

SI.8.5 PEO in DCM and NMF

To estimate the similarity in solvent quality for PEO, shear rheology was also performed in DCM and NMF. As NMF is relatively non-volatile, the zero-shear viscosity of PEO/NMF at 3 mg/mL was measured at 25 °C directly. PEO/NMF behaved as a Newtonian fluid across the measurement range ($\dot{\gamma} \leq 30$ s^{-1}), where η_0 = 8.65 mPa·s. Thus at 3 mg/mL, the specific viscosity for 3 mg/mL PEO/NMF is similar to that for PEO/water and much lower than that for PEO/chloroform (Table S5), suggesting better solvent quality for PEO/chloroform. Despite the substantially higher solvent

viscosity of NMF vs. water, the flow activation energy is lower in NMF (Table S5), supporting RED calculations that NMF is a better solvent (despite similar specific viscosity).

Shear rheology measurements in DCM were more challenging, as PEO demonstrated did not exhibit good solubility in the solvent until 5 °C. Between 5 °C and 10 °C, shear rheology measurements could be performed reproducibly; however, the signal was poorer due to the lower viscosity of PEO/DCM. Above 10 °C, evaporation effects set in; therefore, DCM calculations and temperature-dependent scalings are based on only three points (5, 7.5 and 10 °C, Figure S9).

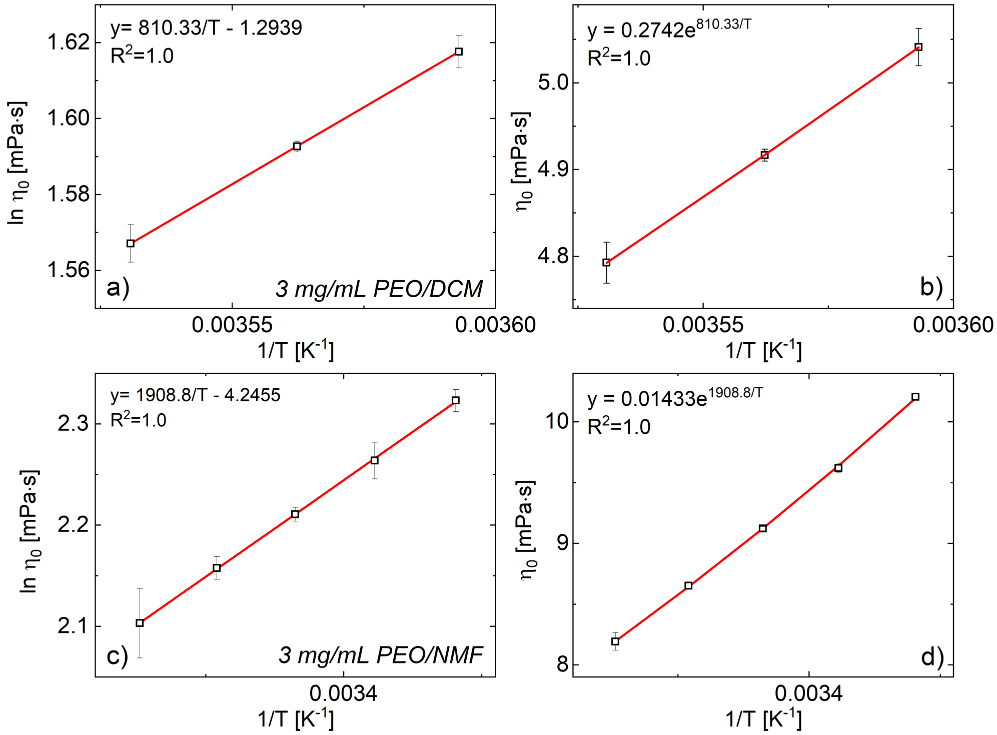

Figure S9: Zero-shear viscosity for 3 mg/mL PEO (1000 kDa) in DCM (a,b) and NMF (c,d) as a function of temperature, which follows an Arrhenius-like dependence, shown in a,c) on a natural log scale (linear fit), and in b,d) on a linear scale (exponential fit).

Using the fit shown in Figure S9, η_0 at 25 °C was estimated to be 4.15 mPa·s for 3 mg/mL PEO/DCM, giving a reduced viscosity of $\eta_{red} = 10.1$ (Table S5). The similar η_{red} for PEO in chloroform and DCM, paired with the fact that their relaxation times scale directly with their solvent viscosities (Table 1) suggests that the Flory exponent and solvent quality is similar, per Eqn. 5. As E_a increases with poorer solvent quality, increasing solution viscosity, and increasing chain stiffness,[13,14] the lower E_a in PEO/DCM vs. PEO/chloroform likely reflects the higher viscosity and thus reduced mobility in chloroform, rather than a difference in solvent quality.

SI.9 Time lapse videos and surface tension of 3 mg/mL PEO in chloroform

To further confirm the film formation during evaporation in PEO/chloroform solutions, time lapse videos were taken on pendant drops for 3 mg/mL PEO in chloroform both in and out of the chamber (Figure S10). Clear evidence of surface film formation and and contraction is observed as the solvent evaporates in Figure S10a (open trials), whereas no inhomogeneities are observed for closed trials in Figure S10b.

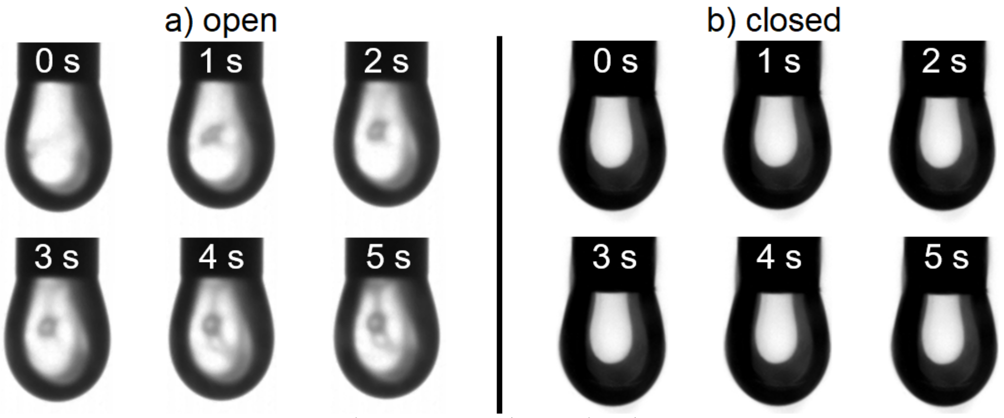

Figure S10: Freely evaporating PEO/chloroform (3 mg/mL) forms a central dimple with radiating wrinkles, distorting the transmitted light. These irregularities are consistent with formation and buckling of a surface skin, as seen in sessile drops of evaporating polymer solutions.[19,20]

To understand differences in evaporation in PEO/chloroform droplets evaporating inside and outside of the chamber, the apparent surface tension during time lapse studies was calculated using drop shape analysis (Figure S11).

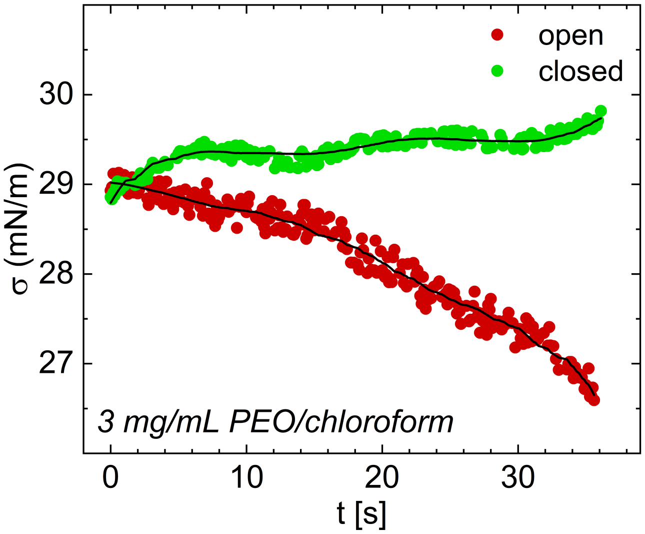

Figure S11: While drop shape analysis is not quantitatively valid for inhomogeneous surfaces, film formation in evaporating PEO/ chloroform leads to a decrease in apparent σ. When t <5 s, no differences in apparent σ are seen between an enclosed (28.78 ± 0.08 mN/m) and freely evaporating drop (28.79 ± 0.13 mN/m) despite visible film formation during this time.

SI.10 Evaporation studies on pendant drops

To assess the efficacy of the environmental control chamber for different solvents, the time to reach 80% of initial drop volume ($t_{0.8}$) was compared between a drop enclosed within the environmental control chamber and a drop open to ambient conditions. The performance of the chamber was calculated in terms of the ratio $\frac{t_{0.8,closed}}{t_{0.8,open}}$. The metric of 80% of initial drop volume was chosen to set a reasonable limit for evaporation, and to ensure that the evaporation rates were constant (linear change in volume with time).

To compare to DoS measurements, evaporation was measured for 3 mg/mL PEO in different solvents, and the chamber was allowed to equilibrate for 45 minutes prior to measurement for closed trials. For the closed trial in water, $t_{0.8}$ was extrapolated from the slope of existing data, and for both trials in NMF, there was virtually no evaporation of the drop. Whereas the environmental control chamber was much more effective in preventing evaporation of DCM than of chloroform, this difference cannot be explained entirely in terms of the equilibration time of the chamber, as $t_{0.8}$ for chloroform increased only to 2.8 minutes after a 2 hour equilibration period, for a performance ratio of 10.5, as shown in SI.11. However, chloroform vapor has a density 4.1× that of air, whereas DCM has a density 2.9× that of air,[3] which suggests that there may be an enriched layer of chloroform vapor at the bottom of the environmental control chamber which slows down chamber equilibration. Based on the minimal gain with longer equilibration times for the chloroform solvent, and that each DoS measurement can be performed in ∼1 minute, a 45 minute equilibration period was chosen.

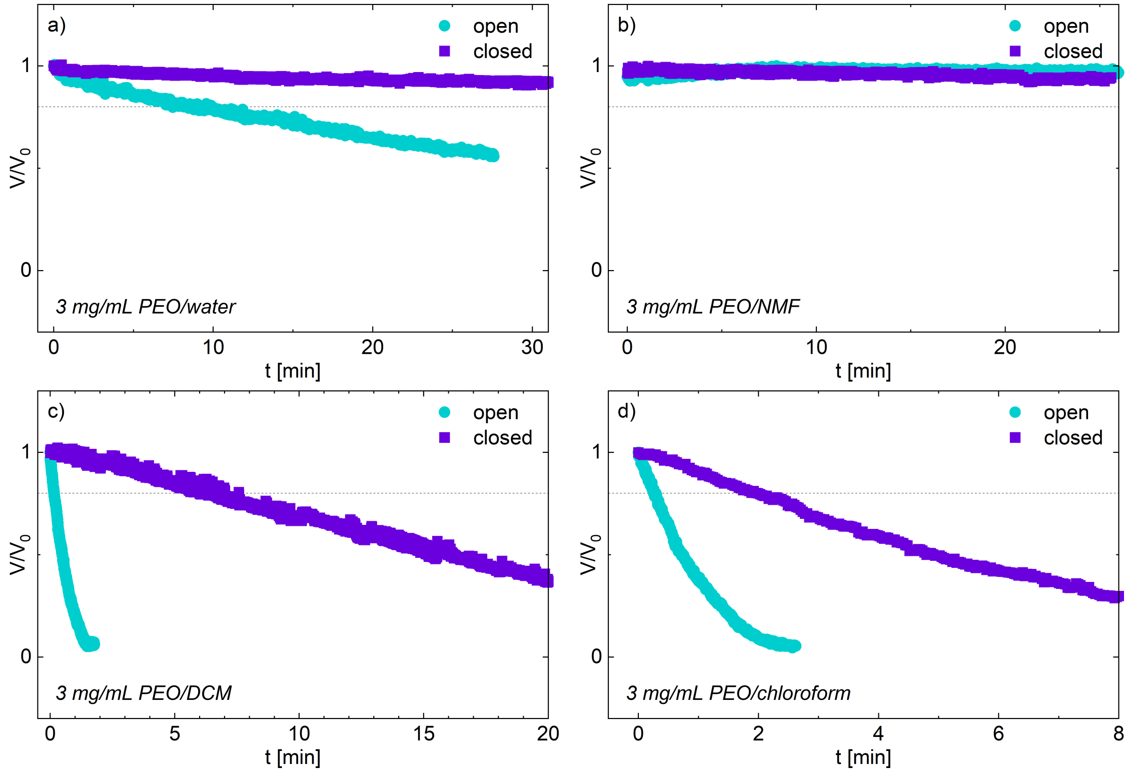

Figure S12: Evaporation of single drops, measured by numerical integration of time-lapse videos and normalized to initial drop size.

To evaluate the effect of equilibration time on droplet evaporation, sequential time-lapse videos were taken of pendant drops of pure chloroform and 3 mg/mL PEO/chloroform every 15 minutes for 2 hours. Normalized volumes of each drop are shown over time in Figure S13.

Although the initial drop size varied from 7 to 10 μL, which led to some variation in the evaporation rate, the first two trials clearly show faster evaporation of the drops after 5 and 15 minutes of equilibration as compared to the drops measured at times later than 30 minutes. This

Table S6: Time for a drop of 3 mg/mL PEO/solvent to evaporate to 80% of its original volume, both inside and outside of the environmental control chamber, and ratio of these evaporation times. The evaporation of PEO/DCM slows by >40x when evaporated inside vs. outside of the chamber.

**solvent**	$t_{0.8,closed}$ **[min]**	$t_{0.8,open}$ **[min]**	**ratio**
chloroform	2.0	0.3	7.2
DCM	6.5	0.2	40.6
NMF	-	-	-
water	83.5	9.3	9.0

trend can be seen more clearly by calculating the average evaporation rates, as in Figure S14.

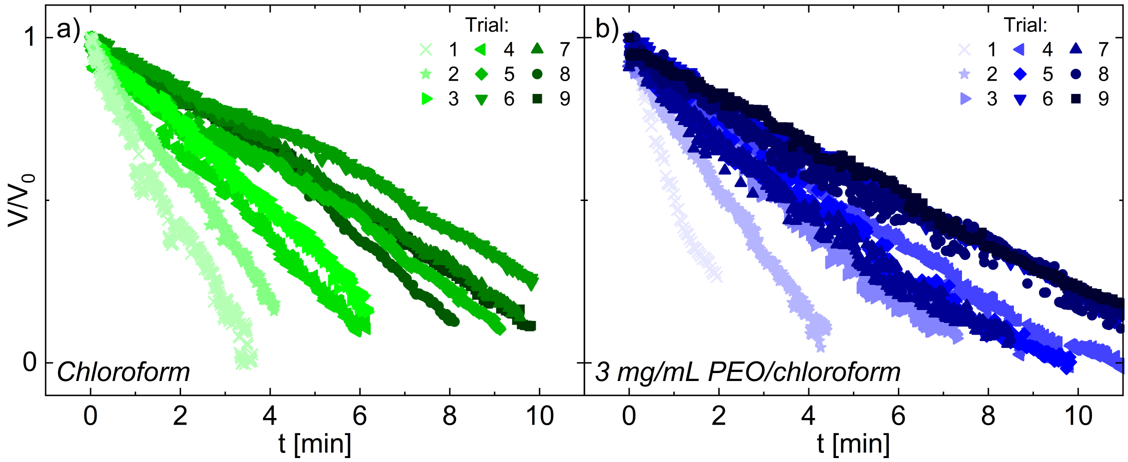

Figure S13: Normalized volumes for nine sequential experiments, performed every 15 minutes for 2 hours, showing evaporation of drops of a) chloroform and b) 3 mg/mL PEO/chloroform

Equilibration occurs very quickly, with evaporation of the PEO solution measured after 3 mins occurring significantly faster than the evaporation of the pure chloroform measured just after 5 minutes. In both cases, evaporation reaches an equilibrium rate after 30 or 45 minutes, meaning that 45 minutes is the correct time to wait for chamber equilibration for chloroform.

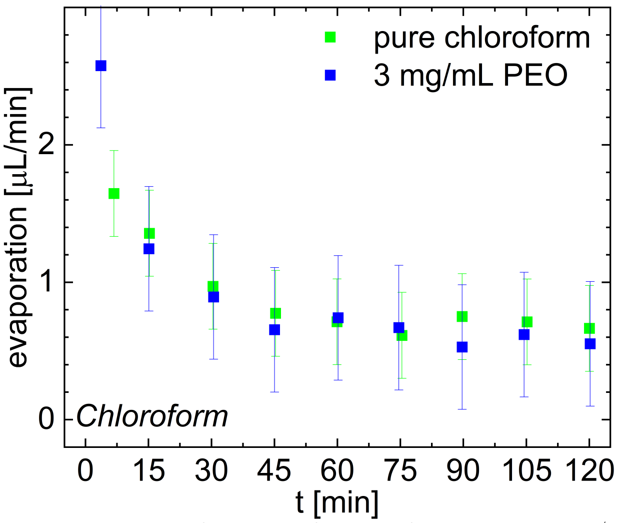

Figure S14: Average evaporation rates of drops of chloroform and 3 mg/mL PEO/chloroform over equilibration time, calculated by linear fits of drop volume over time. Error bars were calculated based on the differences between different fitting regions.

SI.11 Evaporation and extension of chloroform solutions after 2 hours equilibration

To verify the observations made in the sequential time-lapse experiment, the chamber was filled with chloroform and allowed to equilibrate for 2 hours, without intermediate time-lapse experiments to introduce additional solvent vapor into the chamber.

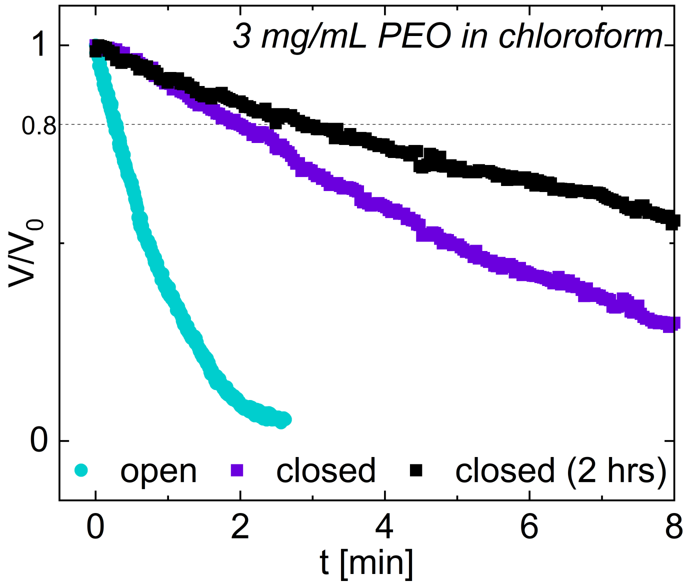

Figure S15: Evaporation of pendant drops of 3 mg/mL PEO/chloroform in different equilibration conditions, measured via time-lapse videos

As shown in Figure S16, the initial evaporation was somewhat slower after 2 hours compared to the standard 45 minutes of equilibration in comparison to free evaporation, with $t_{0.8}$ of 2.8 minutes after 2 hours of equilibration compared to 2 minutes after 45 minutes of equilibration.

Based on these volumes, the concentrations of the drops can be calculated, assuming the drops have a homogeneous composition. Based on evidence from Figure S10, freely evaporating chloroform drops evaporate inhomogeneously, so the open symbols in Figure S16 do not represent actual

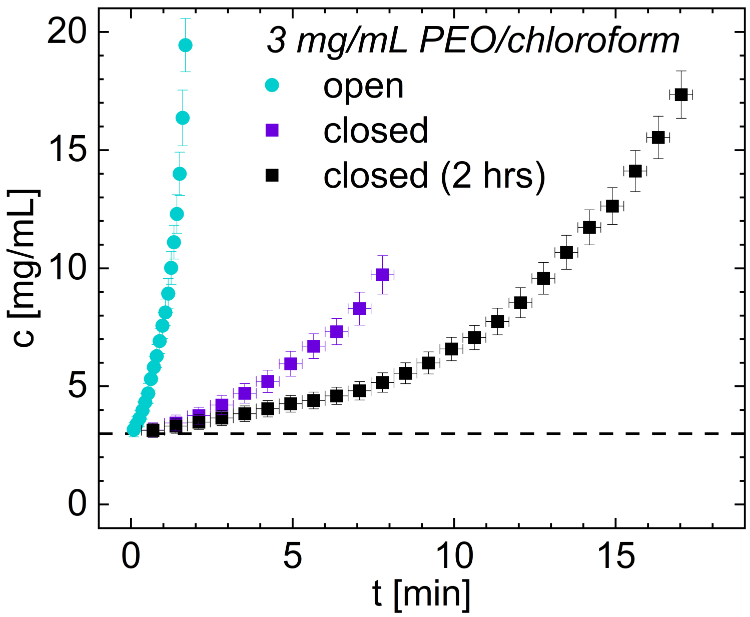

Figure S16: Homogeneous increase in concentration of 3 mg/mL PEO/chloroform solutions due to evaporation, calculated based on a local average volume. Vertical error bars are determined by error propagation of standard deviations of these averages, and horizontal error bars are set as the averaging window of 20 points.

concentrations. Nonetheless, both 45 minutes and 2 hours of equilibration time result in several minutes before the concentration of PEO in initially 3 mg/mL increases homogeneously to 4 mg/mL, allowing measurements of chloroform solutions to be made without large evaporative effects.

As a final experimental verification of the effect of equilibration time on extensional behavior, further DoS trials were performed after 2 hours of equlibration. These trials are shown below in Figure S17 in comparison to the freely evaporating trials and trials after the standard 45 minutes, both of which are also shown in SI.13. Relaxation times, measured from fits to the elasto-capillary region shown via dashed lines, are listed below (Table S7).

Table S7: Relaxation time λ_E extracted from fits to individual trials for 3 mg/mL PEO/chloroform solutions in different equilibration conditions. Average λ_E values reported with standard deviation.

**Sample**	**t1 [ms]**	**t2 [ms]**	**t3 [ms]**	**t4 [ms]**	**t5 [ms]**	**t6 [ms]**	$\lambda_{E,avg}$ **[ms]**
chloroform/open	145	13.4	8.66	185	114	-	93.2 ± 79.2
chloroform/closed (45 min)	4.14	4.71	4.70	4.27	4.24	-	4.41 ± 0.27
chloroform/closed (2 h)	5.24	4.40	4.12	4.15	4.20	4.23	4.39 ± 0.43

Given the nearly identical values of λ_E after 45 minutes ($\lambda_E = 4.41 \pm 0.27$) and 2 hours ($\lambda_E = 4.39 \pm 0.43$) of equilibration, a 45 minute equilibration time was selected.

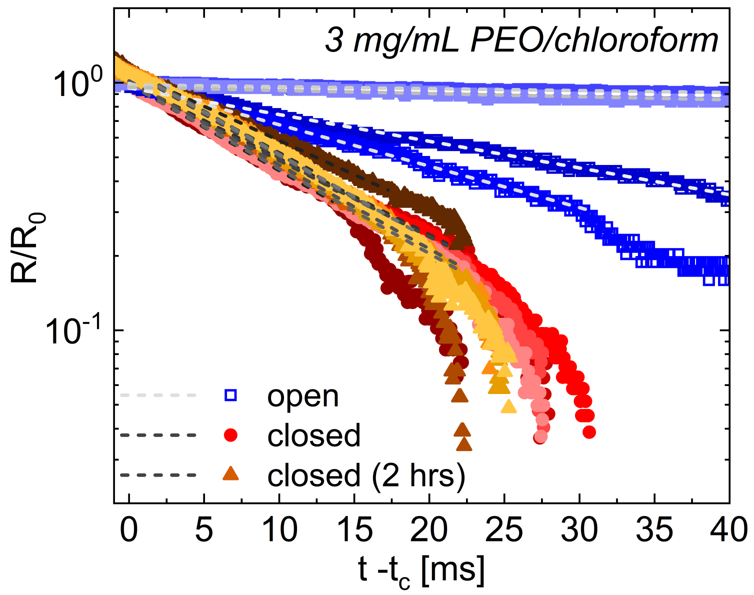

Figure S17: DoS trials for 3 mg/mL PEO/chloroform without environmental control (blue), after 45 minutes of equilibration (red), and after 2 hours of equilibration (yellow). Fits to Eqn. 3 are shown in gray and black dashed lines for open and closed trials, respectively.

SI.12 Estimates of extensional relaxation time for homogeneous concentration

As water and DCM solutions appear to increase in concentration fairly homogeneously, the PEO concentration can be calculated in time similarly to Figure S16, using evaporation data (SI.10).

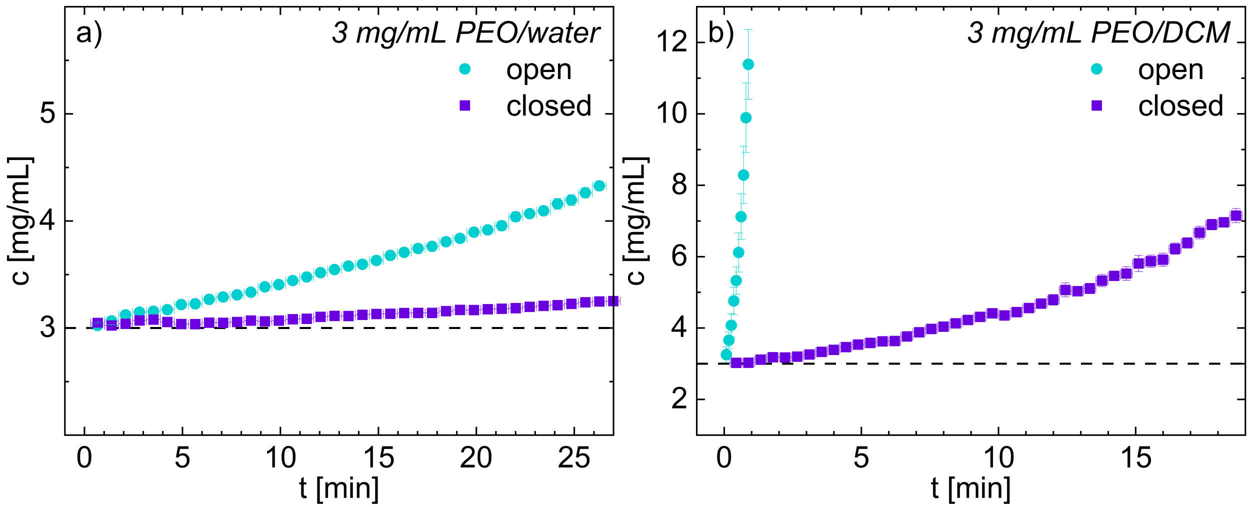

Figure S18: Homogeneous increase in concentration of 3 mg/mL PEO solutions due to evaporation, calculated based on local average volume. Vertical error bars are determined by error propagation of standard deviations of these averages; error bars in t cover the averaging window of 20 points.

These concentrations, and those of chloroform solutions from Figure S16, can then be plugged into Eqn. 5 to extrapolate the expected value of λ_E based on a semi-dilute scaling. This analysis is only valid for small increases in concentration, for which the solutions are still within the semi-dilute regime, but it does allow us to determine how long the drop would have to evaporate for λ_E to be significantly different from the initial measured result (t_{SIG}), as well how long the drop would have

to evaporate for λ_E measured in the chamber to be equal to that of the freely evaporating drop (t_{FREE}). These times are compared in Table S8.

Table S8: Based on the homogeneous concentrations in Figures S16 and S18 and Equation 3, can be extrapolated times (t_{SIG} and t_{FREE}) for λ_E to reach λ_{SIG}, which is significantly larger than λ_E based on a one-tailed t-test (p<0.05), and λ_{FREE}, which is the measured relaxation time of the freely evaporating drop.

**Sample**	t_{SIG} **[s]**	λ_{SIG} **[ms]**	t_{FREE} **[s]**	λ_{FREE} **[ms]**	λ_E **[ms]**
DCM/open	15.73 ± 2.67	4.05 ± 0.28	26.4 ± 2.67	4.65 ± 1.25	3.35 ± 0.17
DCM/closed	399.73 ± 13.33	3.86 ± 0.21	799.73 ± 13.33	4.65 ± 1.25	3.35 ± 0.17
chloroform/open	21.07 ± 2.67	5.25 ± 0.37	-	-	4.41 ± 0.27
chloroform/closed	168.53 ± 21.33	5.44 ± 0.41	-	-	4.41 ± 0.27
chloroform/closed(2 hrs)	211.2 ± 21.33	5.14 ± 0.33	-	-	4.41 ± 0.27
water/open	1107.2 ± 21.33	3.63 ± 0.37	296.53 ± 21.33	3.04 ± 0.24	2.87 ± 0.29
water/closed	-	-	1363.2 ± 21.33	3.04 ± 0.24	2.87 ± 0.29

t_{FREE} can also be used to determine the effective concentration in freely evaporating trials, and thereby evaluate the likelihood of a film. Based on a t_{FREE} of 26.4 seconds for freely evaporating PEO/DCM, the concentration of the open DCM trials (as shown in Figure S18) would be 5.33±0.38 mg/mL. This time corresponds well to the time required to extrude a drop, suggesting that concentration increase rather than film formation raises λ_E during open DoS trials of PEO/DCM.

SI.13 Individual trials and EC fits

Table S9: Relaxation time λ_E extracted from fits to individual trials for all samples. Average λ_E are reported with standard deviation.

**Sample**	**t1 [ms]**	**t2 [ms]**	**t3 [ms]**	**t4 [ms]**	**t5 [ms]**	**t6 [ms]**	$\lambda_{E,avg}$ **[ms]**
NMF/open	4.02	3.53	4.51	4.37	3.43	3.9	3.96 ± 0.43
NMF/closed	3.9	4.05	3.7	3.53	4.51	-	3.94 ± 0.37
water/open	2.92	3.09	3.42	3.01	2.77	-	3.04 ± 0.24
water/closed	2.62	3.25	3.04	2.56	2.87	-	2.87 ± 0.29
DCM/open	3.75	4.92	3.77	4.1	6.73	-	4.65 ± 1.25
DCM/closed	3.53	3.23	3.45	3.13	3.42	-	3.35 ± 0.17
chloroform/open	145	13.4	8.66	185	114	-	93.2 ± 79.2
chloroform/closed	4.14	4.71	4.70	4.27	4.24	-	4.41 ± 0.27

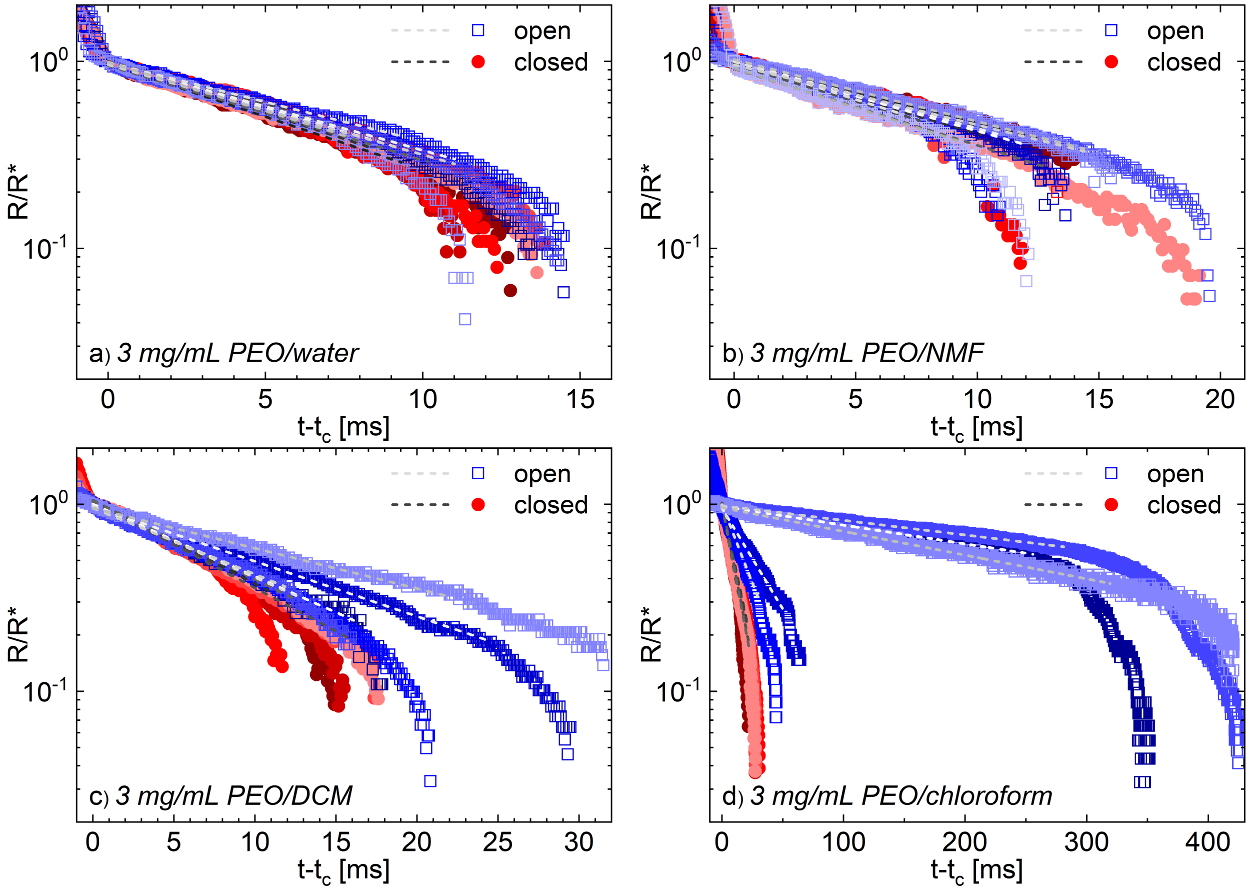

Figure S19: All DoS trials (open and closed) for 3 mg/mL PEO in: a) water, b) NMF, c) DCM, and d) chloroform. Fits to Eqn. 3 are shown in gray and black dashed lines for open and closed trials, respectively.

SI.14 Extension rates and extensional viscosities

Extensional strain rates were calculated for a capillary thinning geometry[21] as $\dot{\varepsilon} = -2\frac{dln(R(t)/R_0)}{dt}$, and plotted as a function of time from the IC/EC transition for representative trials in each solvent and configuration in Figure S20. Extensional viscosities were calculated within the EC regime based on the solution surface tension σ as $\eta_E = \frac{\sigma}{-2dR/dt}$, and plotted against Hencky strain[21], calculated as $\varepsilon = 2ln(\frac{R_0}{R(t)})$, in Figure S21. Extension rates and extensional viscosities are compared for environmentally controlled samples in Figure S23.

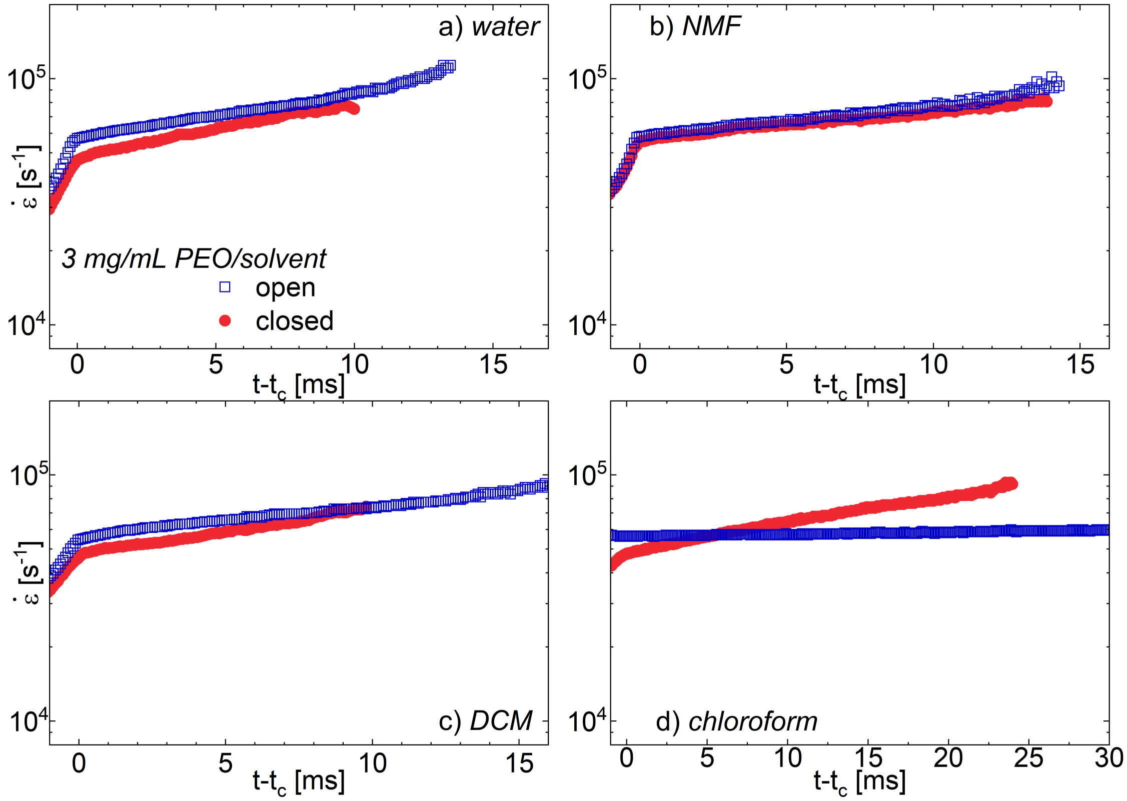

Figure S20: Representative trials depicting extension rates (open and closed) for 3 mg/mL PEO in: a) water, b) NMF, c) DCM, and d) chloroform

All solutions in Figure S20 experience extension rates approaching $\dot{\varepsilon} = 10^5$. With the exception of the open chloroform trial, which has been cropped to account for the greatly longer timescale of thinning, the trends of extension rate over time are very similar between open and closed configurations. Ideal uniaxial extension results in a constant extension rate, so the slight upwards slope of the trials in the EC regime represents a deviation from ideality.

Apparent extensional viscosities within the EC regime are also similar between open and closed configurations at comparable Hencky strains in Figure S21, with the notable exception being chloroform. Note that the differences in ε between open and closed trials or between trials in different solvents occur because of slight variations in R^*. The R^* value varies slightly between trials, and may vary more between PEO trials in different solvents (see Table S2 and Figure S22) and between open vs. closed trials where evaporation is significant. Evaporation effects in chloroform solutions cause an increase in extensional viscosity by over an order of magnitude at comparable strains due to the presence of the surface film.

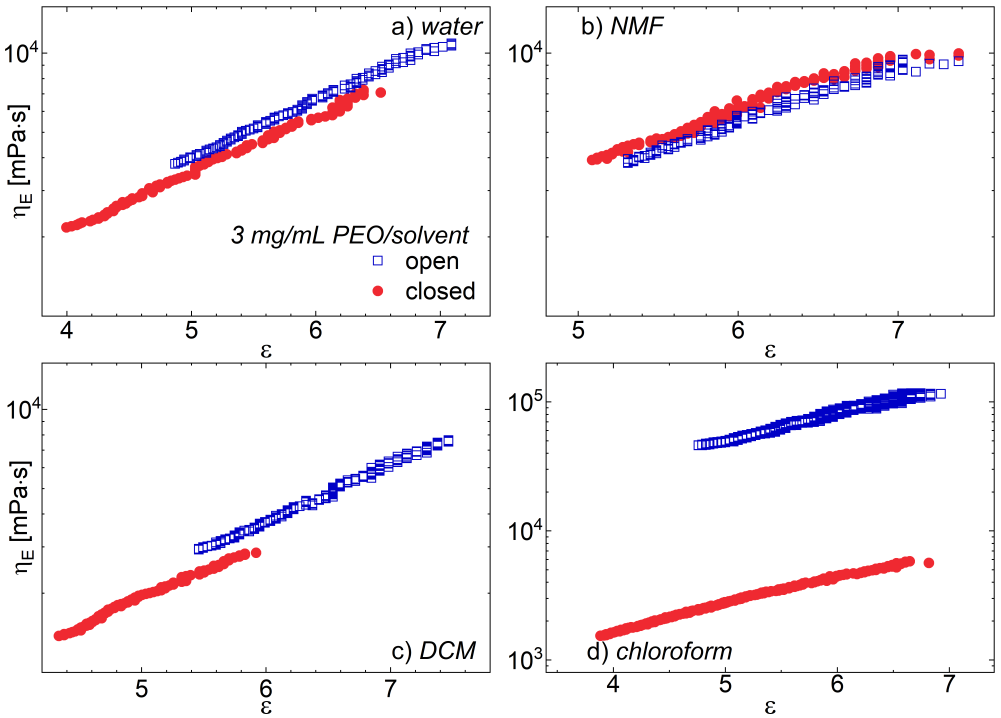

Figure S21: Representative trials depicting extensional viscosities vs. Hencky strains (open and closed) for 3 mg/mL PEO in: a) water, b) NMF, c) DCM, and d) chloroform

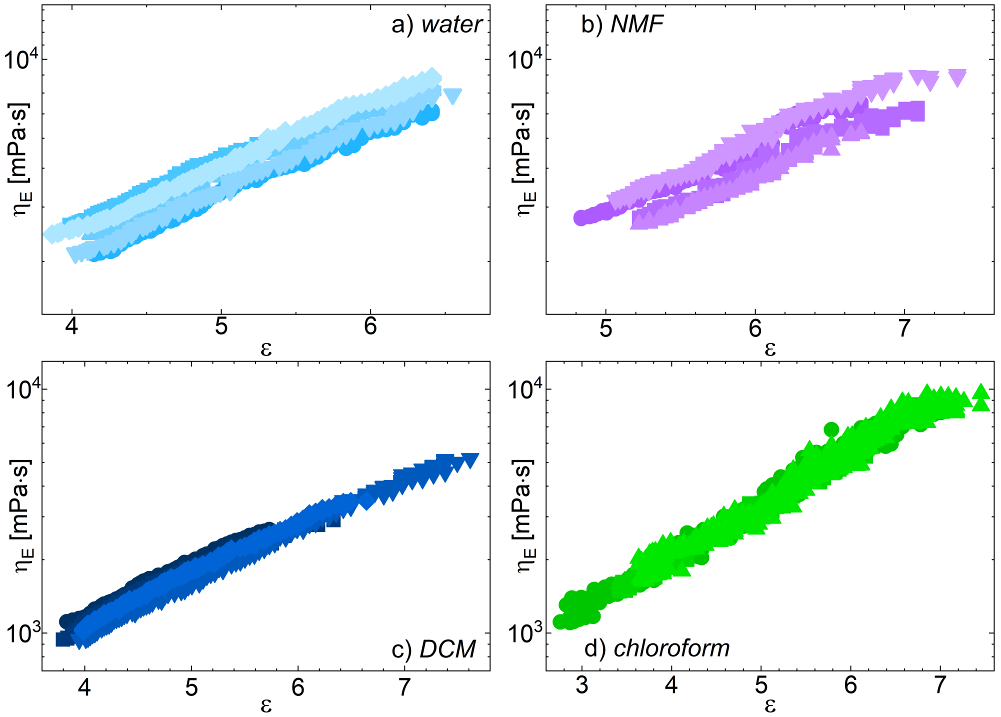

Figure S22: Extensional viscosities vs. Hencky strains of individual trials for 3 mg/mL PEO in: a) water, b) NMF, c) DCM, and d) chloroform.

Whereas extension rates for all solvents are similar when observed under environmental control (Figure S23a), DCM solutions have lower extensional viscosities at similar Hencky strains than all the other samples. Notably, DCM has the lowest solvent viscosity and DCM solutions have the lowest surface tension (see Table 1), directly lowering η_E. Unsurprisingly, when the solution zero-shear viscosity is accounted for (Figure S23b), the Trouton ratios are similar for the three low surface tension solutions (PEO in NMF, DCM and chloroform) but higher for PEO/water, which we attribute to the substantially higher surface tension of water.

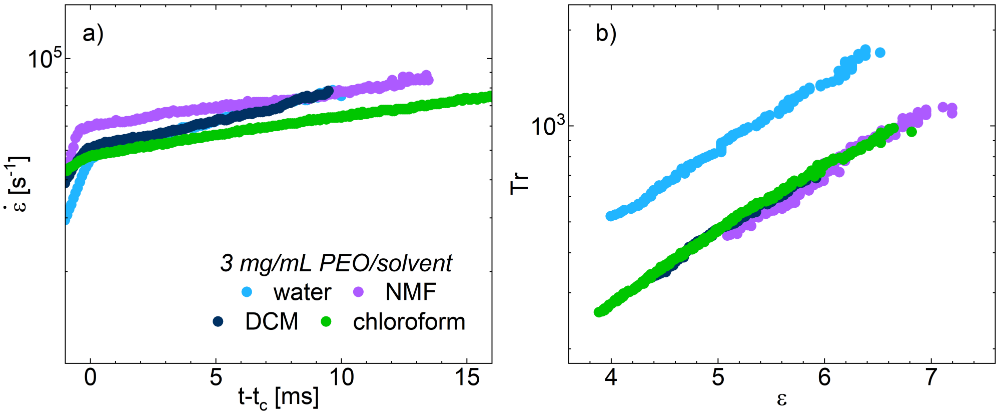

Figure S23: a) Extension rates and b) Trouton ratios $Tr = \eta_E/\eta_0$ for closed trials of 3 mg/mL PEO/solvent.

SI.15 Individual trials and IC fits

For all PEO samples, the estimated Oh is far less than unity; therefore, all PEO samples measured with proper environmental control should display a power law index of $n = 2/3$ immediately prior to the transition to the EC regime. A comparison of at least five trials for each sample taken in the closed chamber are shown in Figures S24-S27 below. Data are represented using $t_f - t$, where t_f is the critical time at the end of the IC regime. All average fits (with uncertainty) encompass the expected IC scaling of $n = 2/3$.

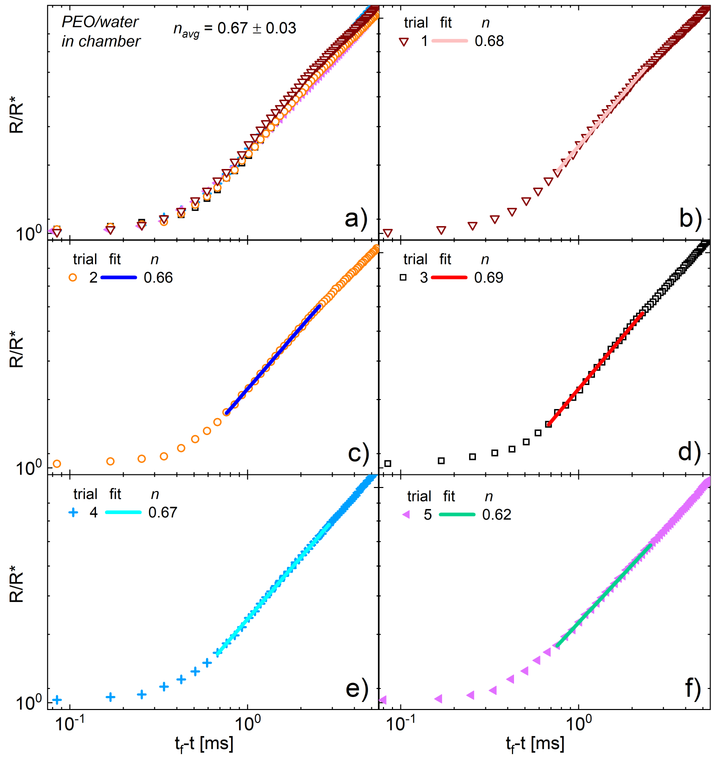

Figure S24: Thinning behavior of 3 mg/mL PEO/water prior to the EC transition. (a) Overlay between five trials. (b-f) Trials and associated fits, giving $n = 0.67 \pm 0.03$.

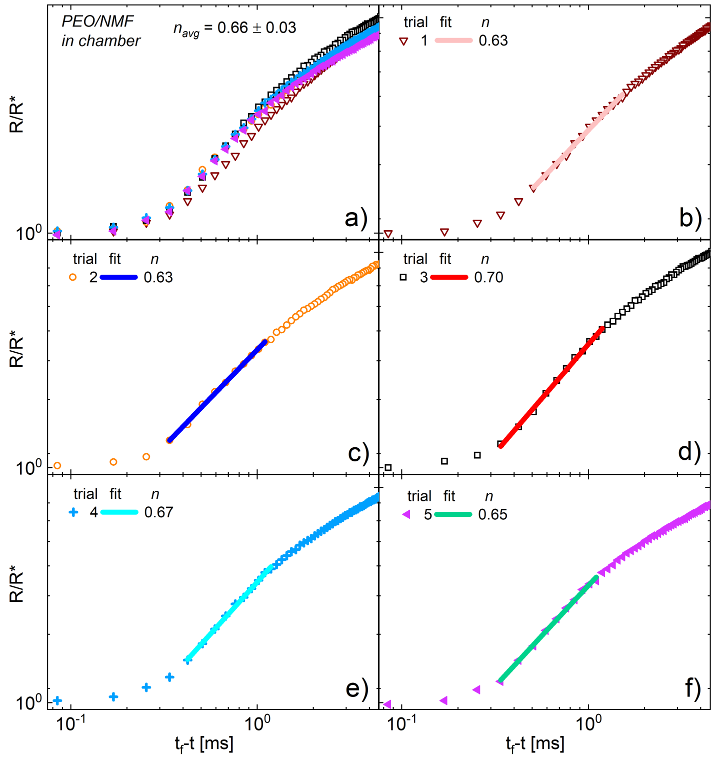

Figure S25: Thinning behavior of 3 mg/mL PEO in NMF prior to the EC transition. (a) Overlay between five trials. (b-f) Individual trials and associated fits, giving $n = 0.66 \pm 0.03$.

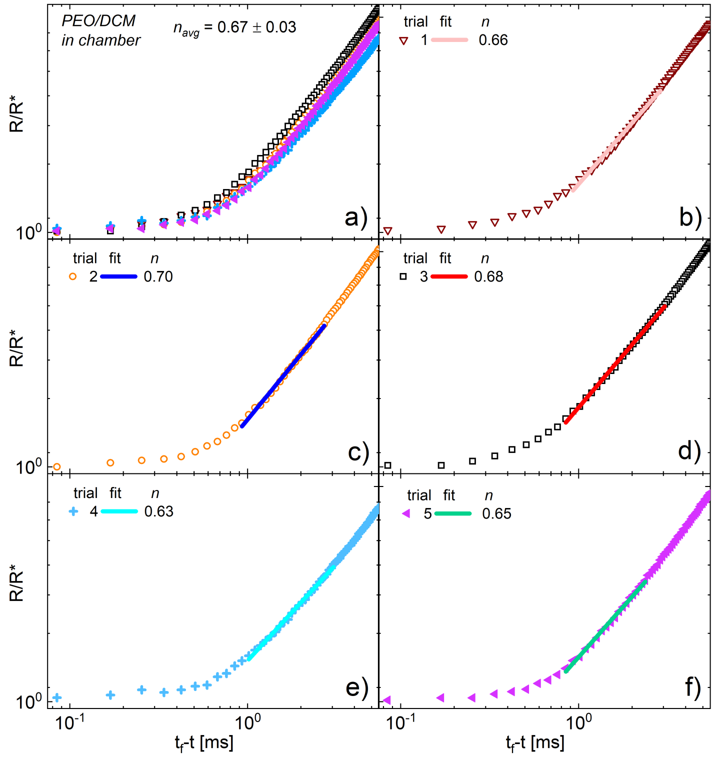

Figure S26: Thinning behavior of 3 mg/mL PEO in DCM prior to the EC transition. (a) Overlay between five trials. (b-f) Individual trials and associated fits, giving $n = 0.67 \pm 0.03$.

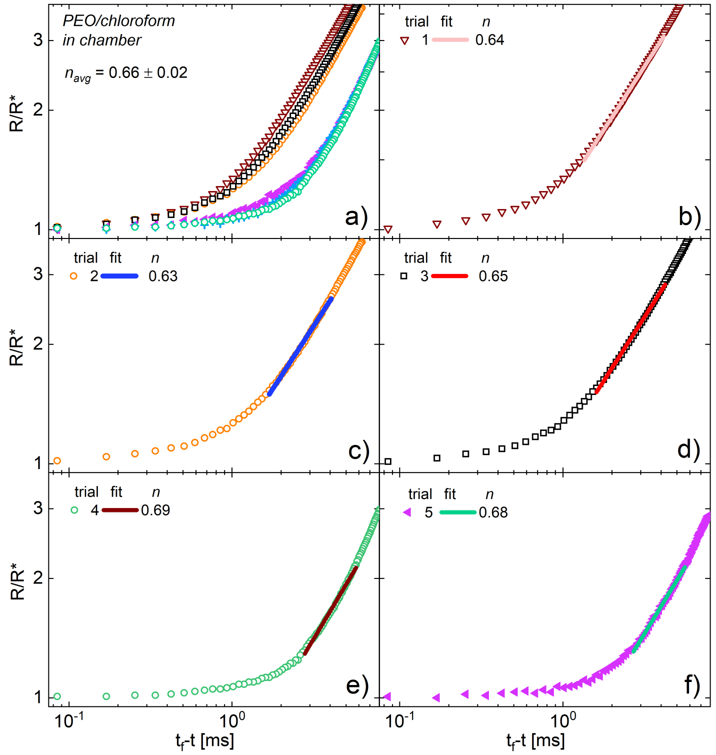

Figure S27: Thinning behavior of 3 mg/mL PEO in chloroform prior to the EC transition. (a) Overlay between 6 trials; groups of trials were taken on the same day. Slight differences in configuration occur during instrument set-up. (b-f) Five individual trials and fits; $n = 0.66 \pm 0.02$.

SI.16 Terminal visco-elasto-capillary (TVEC) fits

Due to the finite extensibility of polymer chains, there is often a terminal visco-elasto-capillary (TVEC) regime that can be captured following the EC regime, described in Equation S7:[21,22]

$$\frac{R}{R_0} = \frac{1}{2\,Oh\,Tr^{\infty}}\left(\frac{t_b - t}{t_R}\right) \tag{S7}$$

where Oh is the Ohnesorge number and t_b is the break-up or pinch-off time, t_R is the Rayleigh time, and Tr^{∞} is the terminal Trouton ratio which is the ratio of the terminal extensional viscosity to the zero-shear viscosity, η_E^{∞}/η_0. Many trials presented in the main text appear to exhibit a TVEC near the end of thinning; this region is fit with Eqn. S7 to obtain η_E^{∞} (Table S10). In Table S10, the maximum extensional viscosity obtained in the same trial is also reported for comparison; we note that because the zero-shear viscosity used to calculate Oh is extrapolated to 25 °C for volatile samples (see SI.8) and is also further interpolated or extrapolated for 2 mg/mL and 5 mg/mL PEO/chloroform, values in Table S10 should be treated as estimates only.

Additionally, the downturn in the radial decay profile corresponding to the TVEC regime can be convoluted with artifacts from poorer resolution near the end of thinning (see SI.1). Briefly, as the filament thins, the contrast against the background is reduced (especially for in-chamber measurements), and points within the liquid bridge become difficult to detect. If this artifact overlaps with the TVEC regime, a steeper slope is observed, thus resulting in a lower viscosity than the true value of η_E^{∞}. As such, the reported values in Table S10 are similar but lower than the maximum η_E seen during the DoS trials, which has been seen in other reports (here η_E^{∞} was as low as $0.8\eta_{E,max}$ in some trials).[23] We also note that in poor lighting conditions, the artifact may be present before the TVEC regime, and the TVEC regime will not be detected.

Table S10: Results for η_E^{∞} from TVEC fits of selected trials depicted in SI.14, compared to maximum values of η_E. The lack of error bars reflects a fit to a single trial. * indicates that η_E^{∞} is less than 80% of $\eta_{E,max}$ and thus likely not indicative of real TVEC behavior. In **, η_0 was extrapolated based on lower concentration data (see SI.7).

Sample	η_E^{∞} [Pa·s]	$\eta_{E,max}$ [Pa·s]
PEO/chloroform (0.5 mg/mL)	1.66*	2.66
PEO/chloroform (1 mg/mL)	1.53	1.80
PEO/chloroform (2 mg/mL)	2.95	3.67
PEO/chloroform (3 mg/mL)	5.26	5.81
PEO/chloroform (5 mg/mL)	2.54**	4.20
PEO/DCM	1.63*	2.47
PEO/NMF	6.10*	9.97
PEO/water	6.71	7.24

To attempt to eliminate this artifact and gain more fitting points in the TVEC region, the threshold may be set to a higher value, but doing so introduces additional noise. The higher threshold should not affect the slope of the points that were detected previously, as rethresholding functionally adds a constant to these values - a portion of the same region can be fit without rethresholding, which results in the same slope and same η_E^{∞}. Depicted in Figure S28 is the best example of TVEC thinning in 3 mg/mL chloroform trials in the chamber, which was obtained by setting the threshold higher during image analysis on the three videos with the highest resolutions. The noise introduced by setting the threshold to a higher than optimal value can be seen in Figure S28, so this was not possible for all trials.

The Trouton ratio obtained from this fit, given $t_R = 5.27$ ms and $Oh = 3.14\cdot 10^{-2}$, was 890. Given

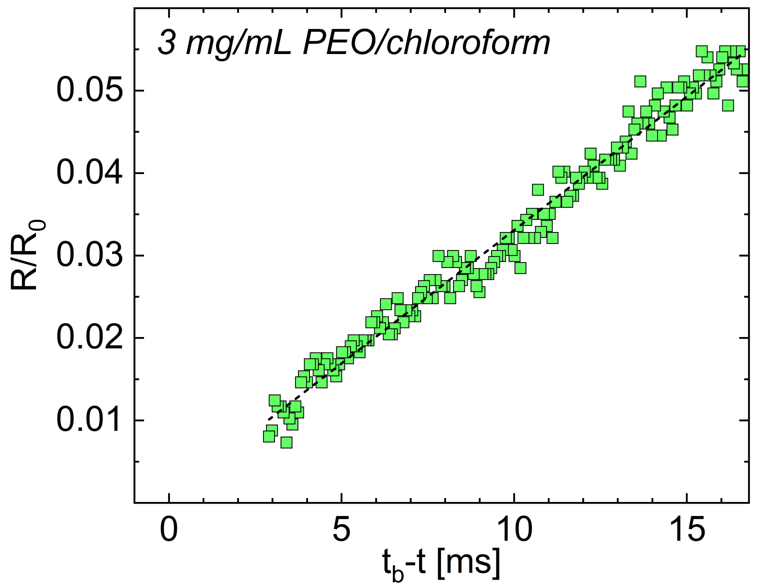

Figure S28: Representative fit of TVEC region to Eqn. S7 after re-thresholding.

the estimated shear viscosity η_0 of 3 mg/mL PEO/chloroform of 5.9 mPa·s (Table S5), a terminal extensional viscosity η_E^∞ of 5.26 Pa·s is obtained whether or not the data was rethresholded, which aligns well with transient extensional viscosities in SI.14.

SI.17 Volume comparisons between methods

DoS uses less sample than most other extensional rheometry methods. For example, plates for Capillary Breakup Extensional Rheometry (CaBER) plates are 4 or 8 mm in diameter. Starting at an initial gap of 3 mm (4 mm plates) or 6 mm (8 mm plates) to produce the same final aspect ratio of 1.5 used in our DoS trials, a volume per trial between 38 and 300 μL is obtained. Practically with loading losses, and given that proper loading requires overfilling, at least 50 to 320 μL are required per trial. Given that we take at least 5 DoS trials using $\sim$10 μL drops to compile accurate statistics, the total difference in volume between the two methods for one sample is 0.2 to 1.55 mL. Assuming an initial cylindrical shape of the liquid bridge, DoS needles with outer diameter 1.63 mm and CaBER plates of diameter 4 or 8 mm, the ratio of surface area to volume is 2.5x to 5x higher in DoS as compared to CaBER.

SI.18 Capillary Breakup Extensional Rheometry (CaBER)

To confirm that evaporation effects were still significant when a sample with a smaller surface area-to-volume ratio was used, Capillary Breakup Extensional Rheometry (CaBER) was performed on 3 mg/mL PEO/chloroform. CaBER was performed with 4 mm plates and strike time of 30 ms, from initial gap of 2 mm to final gap of 4.5 mm. In addition to lengthened breakup times (as was observed during DoS without environmental control), CaBER trials exhibit severe oscillations in the filament diameter; these effects are likely due to inertial effects resulting from the pre-deformation during plate separation.

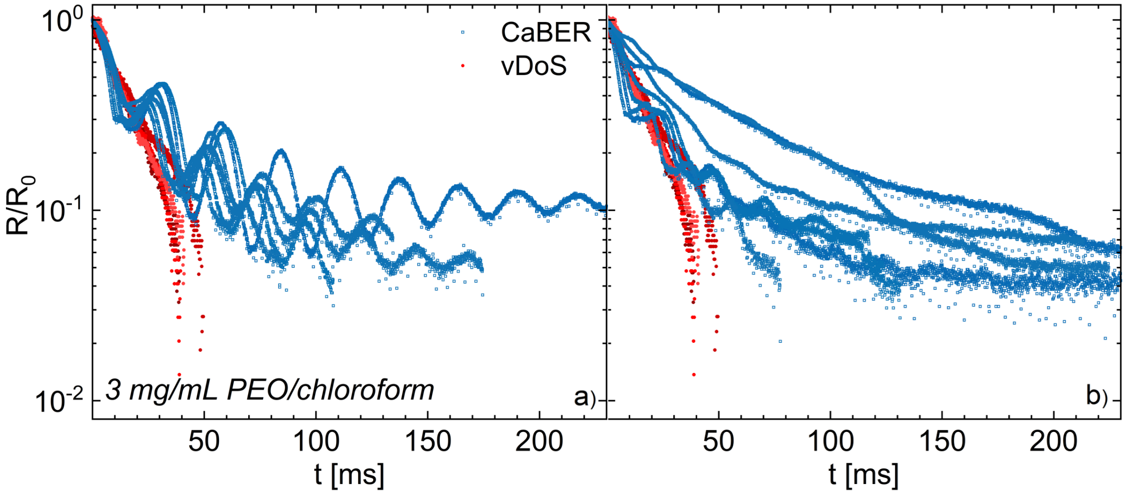

Figure S29: CaBER trials on a 3 mg/mL PEO/chloroform solution. These trials are compared to data extracted from evaporation-controlled DoS - whereas relaxation times measured by DoS are tightly distributed (4.41 ± 0.27 ms), the strong oscillations present in CaBER (a) prevent measurement of relaxation times. However, the slower radial decay for CaBER trials (b) indicates evaporative effects due to the lack of environmental control in a commercial CaBER.

SI.19 Variables and abbreviations

Variables	
a	Mark-Houwink-Sakurada exponent
A	Constant of proportionality for scaling of relaxation time with concentration
b_k	Polymer Kuhn length
c	Concentration
c^*	Critical coil overlap concentration
c_E	Critical entanglement concentration
D	Diffusivity of polymer in solvent
G	Shear modulus
h	Height of liquid bridge in capillary thinning
J	Evaporative flux of solvent per unit area
k	Bolztmann constant
K	Mark-Houwink-Sakurada constant of proportionality
m	Exponent for scaling of relaxation time with concentration
M_W	Weight-average molecular weight
n	Exponent for power-law decay of filament radius
N_k	Kuhn degree of polymerization
Oh	Ohnesorge number
Pe	Peclet number
P_{vap}	Vapor pressure
r_0	Initial size of droplet or liquid bridge
R	Minimum radius of liquid bridge
R^*	Minimum radius of liquid bridge at onset of EC regime
R_0	Outer radius of DoS needle
t	Time
$t_{0.8}$	Time at which 80% of initial drop volume remains
t_b	Characteristic time associated with end of TVEC regime and breakup of filament
t_c	Characteristic time associated with beginning of EC regime
t_f	Characteristic time associated with end of IC regime
t_{FREE}	Time for λ_E inside chamber to reach λ_{FREE} by homogeneous concentration
t_R	Rayleigh time, characteristic thinning time for inviscid fluid
t_{SIG}	Time for λ_E inside chamber to reach λ_{SIG} by homogeneous concentration
T	Temperature
T_{BP}	Boiling point temperature
Tr^∞	Terminal Trouton ratio of η_E^∞ to η_0
z	Vertical coordinate, parallel to DoS needle

Variables (continued)	
α	Constant of proportionality for IC regime
η_0	Solution shear viscosity
$[\eta]$	Intrinsic viscosity
η_s	Solvent shear viscosity
η_{sp}	Specific viscosity
η_{red}	Reduced viscosity
η_E	Apparent extensional viscosity
η_E^∞	Terminal extensional viscosity
λ_E	Extensional relaxation time, as measured during elasto-capillary thinning
λ_{FREE}	Average value of λ_E for freely evaporating drops
λ_{SIG}	Value of λ_E which is significantly higher than the evaporation-controlled λ_E
λ_0	Monomer relaxation time
ν	Flory exponent
ϕ	Volume fraction of polymer
ϕ_0	Initial volume fraction of polymer
ϕ_g	Critical volume fraction of polymer for film formation
ρ	Density
σ	Surface tension

Abbreviations	
CaBER	Capillary Breakup Extensional Rheometry
DCM	Dichloromethane
DoS	Dripping-onto-Substrate
DP	Degree of polymerization
EC	Elasto-capillary
IC	Inertio-capillary
NMF	N-methylformamide
PEO	Polyethylene oxide
RED	Relative Energy Density
TVEC	Terminal visco-elasto-capillary

References

[1] Lauser, K. T., A. Rueter, and M. A. Calabrese, "Small-volume extensional rheology of concentrated protein and protein-excipient solutions," Soft Matter (2021).

[2] Dinic, J., Y. Zhang, L. N. Jimenez, and V. Sharma, "Extensional Relaxation Times of Dilute, Aqueous Polymer Solutions," ACS Macro Lett. **4**, 804 (2015).

[3] "PubChem," (2022), url: https://pubchem.ncbi.nlm.nih.gov/.

[4] "Surface tension values of some common test liquids for surface energy analysis," (2017), url: http://www.surface-tension.de/.

[5] Rodd, L. E., T. P. Scott, J. J. Cooper-White, and G. H. McKinley, "Capillary Break-up Rheometry of Low-Viscosity Elastic Fluids," Applied Rheology **15**, 12 (2005).

[6] Clasen, C., "Capillary breakup extensional rheometry of semi-dilute polymer solutions," Korea-Australia Rheology Journal **22**, 331 (2010).

[7] Hansen, C. M., Hansen solubility parameters: a user's handbook (CRC press, 2007).

[8] Khan, M. S., "Aggregate formation in poly(ethylene oxide) solutions," J. Appl. Polym. Sci. **102**, 2578 (2006).

[9] Chee, M. J. K., J. Ismail, C. Kummerlöwe, and H. W. Kammer, "Study on miscibility of PEO and PCL in blends with PHB by solution viscometry," Polymer **43**, 1235 (2002).

[10] Son, W. K., J. H. Youk, T. S. Lee, and W. H. Park, "The effects of solution properties and polyelectrolyte on electrospinning of ultrafine poly(ethylene oxide) fibers," Polymer **45**, 2959 (2004).

[11] Ebagninin, K. W., A. Benchabane, and K. Bekkour, "Rheological characterization of poly(ethylene oxide) solutions of different molecular weights," Journal of Colloid and Interface Science **336**, 360 (2009).

[12] Eyring, H., "Viscosity, plasticity, and diffusion as examples of absolute reaction rates," J. Chem. Phys. **4**, 283 (1936).

[13] Tager, A., V. Y. Dreval, and F. Khasina, "Concentrated polymer solutions—iii. viscosity of polyisobutylene solutions in various solvents," Polymer Science USSR **4**, 1097 (1963).

[14] Yokotsuka, S., Y. Okada, Y. Tojo, T. Sasaki, and M. Yamamoto, "Activation energy of local polymer motions estimated from the fluorescence depolarization measurements," Polymer J. **23**, 95 (1991).

[15] Hiemenz, P. C., and T. P. Lodge, "Dynamics of DIlute Polymer Solutions," in Polymer Chemistry (CRC Press, 2007) 2nd ed.

[16] Dinic, J., M. Biagioli, and V. Sharma, "Pinch-off dynamics and extensional relaxation times of intrinsically semi-dilute polymer solutions characterized by dripping-onto-substrate rheometry," J. Polym. Sci. B Polym. Phys. **55**, 1692 (2017).

[17] Ferry, J. D., E. L. Foster, G. V. Browning, and W. Sawyer, "Viscosities of concentrated polyvinyl acetate solutions in various solvents," J. Colloid Sci. **6**, 377 (1951).

[18] Yasuda, K., R. Armstrong, and R. Cohen, "Shear flow properties of concentrated solutions of linear and star branched polystyrenes," Rheologica Acta **20**, 163 (1981).

[19] Pauchard, L., and C. Allain, "Stable and unstable surface evolution during the drying of a polymer solution drop," Physical Review E **68**, 052801 (2003).

[20] Okuzono, T., K. Ozawa, and M. Doi, "Simple model of skin formation caused by solvent evaporation in polymer solutions," Phys. Rev. Lett. **97**, 136103 (2006).

[21] McKinley, G. H., "Visco-elasto-capillary thinning and break-up of complex fluids," Rheology Rev. (2005).

[22] Dinic, J., and V. Sharma, "Macromolecular relaxation, strain, and extensibility determine elastocapillary thinning and extensional viscosity of polymer solutions," Proc Natl Acad Sci USA **116**, 8766 (2019).

[23] Dinic, J., and V. Sharma, "Flexibility, Extensibility, and Ratio of Kuhn Length to Packing Length

Govern the Pinching Dynamics, Coil-Stretch Transition, and Rheology of Polymer Solutions," Macromolecules (2020).
